# Randomized controlled trials in de-implementation research: a systematic scoping review

**DOI:** 10.1186/s13012-022-01238-z

**Published:** 2022-10-01

**Authors:** Aleksi J. Raudasoja, Petra Falkenbach, Robin W. M. Vernooij, Jussi M. J. Mustonen, Arnav Agarwal, Yoshitaka Aoki, Marco H. Blanker, Rufus Cartwright, Herney A. Garcia-Perdomo, Tuomas P. Kilpeläinen, Olli Lainiala, Tiina Lamberg, Olli P. O. Nevalainen, Eero Raittio, Patrick O. Richard, Philippe D. Violette, Jorma Komulainen, Raija Sipilä, Kari A. O. Tikkinen

**Affiliations:** 1 Faculty of Medicine, University of Helsinki, Helsinki, Finland; 2 Finnish Medical Society Duodecim, Helsinki, Finland; 3 Finnish Coordinating Center for Health Technology Assessment, Oulu University Hospital, Oulu, Finland; 4 University of Oulu, Oulu, Finland; 5 Department of Nephrology and Hypertension, University Medical Center Utrecht, Utrecht University, Utrecht, The Netherlands; 6 Julius Center for Health Sciences and Primary Care, University Medical Center Utrecht, Utrecht University, Utrecht, The Netherlands; 7 Occupational Health Helsinki, Helsinki, Finland; 8 Division of General Internal Medicine, Department of Medicine and Department of Health Research Methods, Evidence and Impact, McMaster University, Hamilton, ON Canada; 9 Department of Urology, University of Fukui Faculty of Medical Sciences, Fukui, Japan; 10 Department of General Practice and Elderly Care Medicine, University Medical Centre Groningen, University of Groningen, Groningen, The Netherlands; 11 Department of Gynaecology, Chelsea & Westminster NHS Foundation Trust, London, UK; 12 Department of Epidemiology & Biostatistics, Imperial College London, London, UK; 13 Division of Urology/Uro-oncology, Department of Surgery, School of Medicine, Universidad del Valle, Cali, Colombia; 14 Department of Urology, University of Helsinki and Helsinki University Hospital, Helsinki, Finland; 15 Department of Radiology, Tampere University Hospital and Faculty of Medicine and Health Technologies, Tampere University, Tampere, Finland; 16 Hatanpää Health Center, City of Tampere, Finland; 17 Unit of Health Sciences, Faculty of Social Sciences, Tampere University, Tampere, Finland; 18 Oral Health Care, Tampere, Finland; 19 Institute of Dentistry, University of Eastern Finland, Kuopio, Finland; 20 Nordic Healthcare Group Ltd., Helsinki, Finland; 21 Division of Urology, Centre Hospitalier Universitaire de Sherbrooke, Sherbrooke, Canada; 22 Departments of Surgery and Health Research Methods Evidence and Impact, McMaster University, Hamilton, Canada; 23 Department of Surgery, South Karelian Central Hospital, Lappeenranta, Finland

**Keywords:** Clinical trials, Cluster randomized trial, De-implementation, Methods, Overuse, Randomized controlled trial, Scoping review, Trial design, Low-value care

## Abstract

**Background:**

Healthcare costs are rising, and a substantial proportion of medical care is of little value. De-implementation of low-value practices is important for improving overall health outcomes and reducing costs. We aimed to identify and synthesize randomized controlled trials (RCTs) on de-implementation interventions and to provide guidance to improve future research.

**Methods:**

MEDLINE and Scopus up to May 24, 2021, for individual and cluster RCTs comparing de-implementation interventions to usual care, another intervention, or placebo. We applied independent duplicate assessment of eligibility, study characteristics, outcomes, intervention categories, implementation theories, and risk of bias.

**Results:**

Of the 227 eligible trials, 145 (64%) were cluster randomized trials (median 24 clusters; median follow-up time 305 days), and 82 (36%) were individually randomized trials (median follow-up time 274 days). Of the trials, 118 (52%) were published after 2010, 149 (66%) were conducted in a primary care setting, 163 (72%) aimed to reduce the use of drug treatment, 194 (85%) measured the total volume of care, and 64 (28%) low-value care use as outcomes. Of the trials, 48 (21%) described a theoretical basis for the intervention, and 40 (18%) had the study tailored by context-specific factors. Of the de-implementation interventions, 193 (85%) were targeted at physicians, 115 (51%) tested educational sessions, and 152 (67%) multicomponent interventions. Missing data led to high risk of bias in 137 (60%) trials, followed by baseline imbalances in 99 (44%), and deficiencies in allocation concealment in 56 (25%).

**Conclusions:**

De-implementation trials were mainly conducted in primary care and typically aimed to reduce low-value drug treatments. Limitations of current de-implementation research may have led to unreliable effect estimates and decreased clinical applicability of studied de-implementation strategies. We identified potential research gaps, including de-implementation in secondary and tertiary care settings, and interventions targeted at other than physicians. Future trials could be improved by favoring simpler intervention designs, better control of potential confounders, larger number of clusters in cluster trials, considering context-specific factors when planning the intervention (tailoring), and using a theoretical basis in intervention design.

**Registration:**

OSF Open Science Framework hk4b2

**Supplementary Information:**

The online version contains supplementary material available at 10.1186/s13012-022-01238-z.

Contributions to the literature

- Our systematic scoping review gives the first comprehensive overview of randomized controlled trials in de-implementation.
- De-implementation trials have focused on primary care and drug treatments; however, there is dire lack of research on diagnostics, surgical treatments, and in secondary/tertiary care.
- Most trials were limited by complex intervention design, human intervention deliverer, small number of clusters in cluster trials, and lack of theoretical background and tailoring.
- Major improvements in methodology are needed to find reliable evidence on effective de-implementation interventions. We provided recommendations on how to address these issues.

## Introduction

Despite rising appreciation of evidence-based practices, current medical care is often found to be of low value for patients [1]. Low-value care has been described as care that (i) provides little or no benefit, (ii) potentially causes harm, (iii) incurs unnecessary costs to patients, or (iv) wastes healthcare resources [2]. After the adoption of low-value care practices, abandoning them is often difficult [3, 4]. This might be due to several psychological reasons, including fear of malpractice, patient pressures, and “uncertainty on what not to do” [5, 6].

With constantly rising healthcare costs, allocating resources in ways that provide the best benefit for the patients is very important. De-implementation — strategies to reduce low-value care use — is an important part of future healthcare planning. Four types of de-implementation have been described: (i) removing, (ii) replacing, (iii) reducing, or (iv) restricting care [7]. As de-implementation interventions aim to induce behavioral change with numerous factors affecting the outcome, both the research environment and methodology are complex [7]. Thus, high-quality randomized controlled trials (RCTs) are needed to reliably estimate the effect of different strategies [8].

Despite the increasing number of published de-implementation RCTs, there are no previous comprehensive systematic or scoping reviews summarizing the de-implementation RCTs. We conducted a systematic scoping review to map the current state of de-implementation research, including potential knowledge gaps and priority areas. We also aimed to provide guidance for future researchers on how to provide trustworthy evidence.

## Methods

We performed a systematic scoping review, registered the protocol in Open Science Framework (OSF hk4b2) [9], and followed the Preferred Reporting Items for Systematic reviews and Meta-Analyses extension for Scoping Reviews (PRISMA-ScR) checklist [10] (Additional file 2).

### Data sources and searches

We developed a comprehensive search strategy in collaboration with an experienced information specialist (T. L.) (Additional file 1, eMethods 1). We searched MEDLINE and Scopus for individual and cluster RCTs of de-implementation interventions without language limits through May 24, 2021. First, we used terms identified by an earlier scoping review of de-implementation literature [11] (judged useful in earlier de-implementation research [12, 13]). Second, we identified relevant articles from previously mentioned [11] and two other [3, 4] earlier systematic reviews of de-implementation. Using these identified articles, we updated our search strategy with new index terms (Additional file 1, eMethods 1). Third, we performed our search with all identified search terms (step 1 and step 2). Fourth, we identified systematic reviews (found by our search) and searched their reference lists for additional potentially eligible articles. Finally, we followed up protocols and post hoc analyses (identified by our search) of de-implementation RCTs and added their main articles to the selection process.

### Eligibility criteria

We included all types of de-implementation interventions across all medical specialties. We included trials comparing a de-implementation intervention to a placebo, another de-implementation intervention, or usual care. We included studies with any target group, including patients with any disease as well as all kinds of healthcare professionals, organizations, and laypeople. In our review, we excluded deprescribing trials as we considered the context of stopping a treatment already in use (deprescribing) to be somewhat different than the context of not starting a certain treatment (de-implementation), for example, stopping use of long-term benzodiazepines for anxiety disorders (deprescribing) vs not starting antibiotics for viral respiratory tract infections (de-implementation) [14]. We also excluded trials only aiming to reduce resource use (e.g., financial resources or clinical visits) and trials where a new medical practice, such as laboratory test, was as an intervention to reduce the use of another practice.

### Outcomes and variables

We collected and evaluated the following outcomes/variables: (1) study country, (2) year of publication, (3) unit of randomization allocation (individual vs. cluster), (4) the number of clusters, (5) was an intra-cluster correlation (ICC) used in sample size calculation, (6) duration of follow-up, (7) setting, (8) medical content area, (9) target group for intervention, (10) the number of study participants, (11) mean age of study participants, (12) the proportion of female participants, (13) intervention categories, (14) rationale for de-implementation, (15) goal of the intervention, (16) outcome categories, (17) reported effectiveness of the intervention, (18) conflicts of interest, (19) funding source, (20) risk of bias, (21) implementation theory used, (22) costs of the de-implementation intervention, (23) effects on total healthcare costs, (24) changes between baseline and after the intervention, and (25) tailoring the de-implementation intervention to study context.

### Risk of bias and quality indicators

To improve judgements regarding the studies with complex intervention designs and to enhance the interrater agreement [15] in risk-of-bias assessment, through iterative discussion, consensus building, and informed by previous literature [16, 17], we modified the Cochrane risk-of-bias tool for cluster randomized trials [18] (Additional file 1, eMethods 2). Studies were rated based on six criteria: (1) randomization procedure, (2) allocation concealment, (3) blinding of outcome collection, (4) blinding of data analysts, (5) missing outcome data, and (6) imbalance of baseline characteristics. For each criterion, studies were judged to be at either high or low risk of bias. In addition, we collected data on the number of clusters, length of follow-up, intra-cluster correlation, tailoring, theoretical background, level of randomization, and reported differences before and after the baseline, and considered these as quality indicators.

### Study selection and data extraction

We developed standardized forms with detailed instructions for screening abstracts and full texts, risk of bias assessment, and data extraction (including outcomes/variables, intervention categorization, and outcome hierarchy). Independently and in duplicate, two methodologically trained reviewers applied the forms to screen study reports for eligibility and extracted data. Reviewers resolved disagreements through discussion and, if necessary, through consultation with a clinician-methodologist adjudicator.

### Intervention categorization and outcome hierarchy

To define categories for the rationale of de-implementation, we used a previous definition of low-value care: “care that is unlikely to benefit the patient given the harms, cost, available alternatives, or preferences of the patient” [2].

We modified the Effective Practice and Organisation of Care (EPOC) taxonomy of health systems interventions to better fit the current de-implementation literature [19]. First, we categorized the interventions from eligible studies according to the existing EPOC taxonomy. Second, we discussed the limitations of the EPOC taxonomy with our multidisciplinary team and built consensus on modifications (categories to be modified, excluded, divided, or added). Finally, we repeated the categorization by using our refined taxonomy. Disagreements were solved by discussion and/or by consulting an implementation specialist adjudicator. Full descriptions of intervention categories and the rationale for the modifications are available in the Additional file 1 (eMethods 3 and 4).

To develop outcome categories for effectiveness outcomes (Table 1), we modified Kirkpatrick’s levels for educational outcomes [20]. We identified five categories: health outcomes, low-value care use, appropriate care use, total volume of care, and intention to reduce low-value care. A complete rationale for the hierarchy of outcomes is available in the Additional file 1 (eMethods 5).

**Table 1:** Outcome categories for de-implementation effectiveness

Name	Rationale and definitions	Examples
Health outcomes	De-implementing a clinical practice should improve (or at least have no negative effect on) health outcomes. Health outcomes can therefore be considered measuring the safety of de-implementation	Mortality, morbidity, quality of life, symptoms
Low-value care use	The primary aim of a de-implementation intervention is to reduce low-value care. Predefined low-value care use should therefore be (one of) the primary outcome(s) of de-implementation effectiveness. Typically, the definition of low-value care is based on diagnoses or clinical criteria that represent low-value care in combination with a specific clinical practice. Data is often gathered from individual patient records or administrative databases. Individual patient records usually contain more specific information on clinical decisions and may therefore yield more accurate information	Antibiotic use for viral upper respiratory infections Use of radiological imaging in patients with acute low back pain without “red-flag” symptoms
Appropriate care use	Can be used as an outcome when a medical practice can be either appropriate or inappropriate. For instance, in patients with respiratory infection, use of antibiotics can be either appropriate or inappropriate. Change in appropriate care use measures unintended consequences of de-implementation and can therefore be considered as a measure of safety of de-implementation	Antibiotic use for confirmed pneumonia Use of radiological imaging in patients with low back pain and “red-flag” symptoms
Total volume of care	Total volume includes both appropriate and inappropriate care and is an indirect measure of low-value care. It may sometimes be justifiable to use in very large samples if it is impossible to differentiate between appropriate and inappropriate care and if using individual patient records is not possible. Outcomes that are based on diagnoses often include both appropriate and inappropriate care and should therefore be considered as total volume care, not as low-value care, outcomes	Total use of antibiotics in upper respiratory tract infections Use of radiological imaging in low-back pain
Intention to reduce the use of low-value care	Intention is the first step to change but does not reliably describe actual change in use of low-value care. As intention can be measured earlier than other outcomes, it may sometimes be justifiable to use as a preliminary assessment of the effectiveness of a de-implementation intervention. It is often used after educational interventions and when the data is gathered through surveys	Intention to reduce the use of inappropriate antibiotic use in upper respiratory tract infections Intention to reduce use of inappropriate radiological imaging in low-back pain

### Analysis

We used summary statistics (i.e., frequencies and proportions, typically with interquartile ranges) to describe study characteristics. We compared quality indicators (see paragraph “Risk of bias and quality indicators”) between studies published in 2010 or before and after 2010 to explore potential changes in trial methodology and execution. Finally, considering the lack of methodological standards in de-implementation literature (also identified by our scoping review), we created recommendations for future de-implementation research. Through discussion and consensus building, we drafted recommendations in several in-person meetings. Subsequently, authors gave feedback on the drafted recommendations by email. Finally, we made final recommendations in in-person meetings.

## Results

We screened 12,815 abstracts, of which 1025 articles were potentially eligible. After screening full texts, 204 articles were included in the data extraction. In addition, we included 31 articles from hand-searching of references of systematic review and 5 articles from study protocols and post hoc analyses. In total, we identified 240 published articles from 227 unique studies (PRISMA flow diagram in the Additional file 1, eFig. 1).

### Study characteristics

**Fig. 1:**
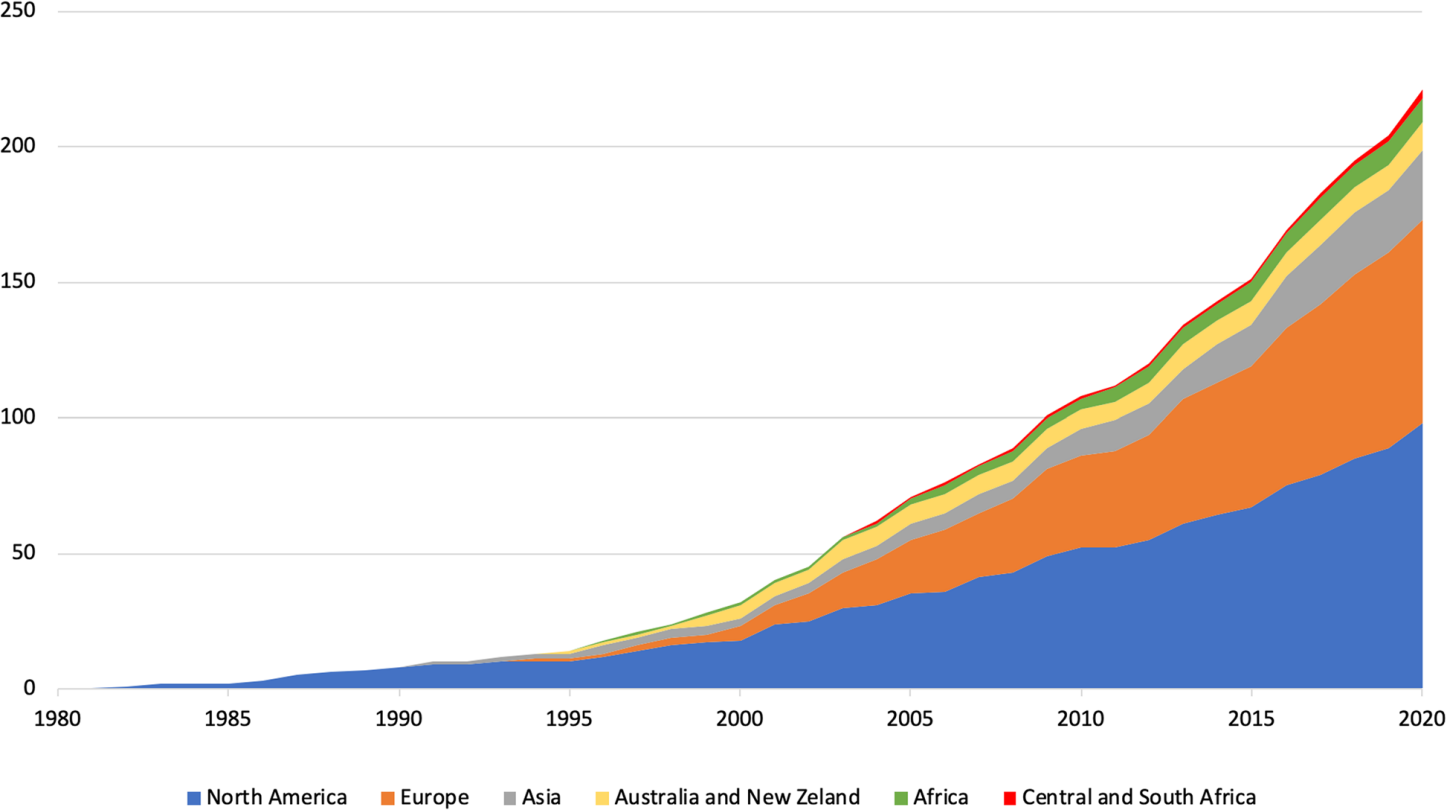
Published de-implementation randomized controlled trials over time, stratified by continent

Studies were published between 1982 and 2021; half of them were published in 2011 or later. Of the 227 trials identified, 44% (*n* = 101) were conducted in North America (of which 83 in the USA), 33% (*n* = 76) in Europe, and the rest in other regions (Fig. 1). Of the 227 trials, 145 (64%) used a cluster design and 82 (36%) an individually randomized design; 149 (66%) were conducted in primary care and 65 trials (29%) in secondary or tertiary care (Table 2).

**Table 2:** Description of the included 227 randomized controlled trials: characteristics, aims, and outcomes

Characteristics		Aim and rationale		Outcomes	
*Setting*^*a*^	*n* (%)	*Aim*^*a*^	*n* (%)	*Outcome categories*^*a*^	*n* (%)
Primary care — outpatient	149 (66%)	Abandon	0 (0%)	Health outcomes	58 (26%)
Primary care — inpatient	3 (1%)	Reduce	225 (99%)	Low-value care use	63 (28%)
Secondary/tertiary care — outpatient	28 (12%)	Replace	42 (19%)	Appropriate care use	34 (15%)
Secondary/tertiary care — inpatient	40 (18%)	Unclear	2 (1%)	Total volume of care	194 (85%)
Other	22 (10%)	*Rationale*^*a*^		Intention to reduce the use of low-value care	
*Randomization unit*		Evidence suggests little or no benefit from treatment or diagnostic test	115 (51%)		17 (7%)
Cluster	145 (64%)			*Measured costs*^*a*^	
Individual	82 (36%)	Evidence suggests another treatment is more effective or less harmful	13 (6%)	Intervention costs	20 (9%)
*Medical intervention*^*a*^				Healthcare costs	45 (20%)
Prevention	9 (4%)	Evidence suggests more harms than benefits for the patient or community	145 (64%)	*Reported effectiveness*	
Diagnostic imaging	29 (13%)			(Some) desired effect	186 (82%)
Laboratory tests	28 (12%)	Cost-effectiveness	70 (31%)	No desired effect	41 (18%)
Drug treatment	163 (72%)	Patient(s) do not want the intervention	2 (1%)	*Theoretical basis and tailoring*^*a*^	
Operative treatments	7 (3%)			Theory-based interventions	48 (21%)
Rehabilitation	2 (1%)	Not reported/unclear	20 (9%)	Tailored interventions	40 (18%)
Other	7 (3%)			*Intervention complexity*^*b*^	
*Target group*^*a*^				Multicomponent	152 (67%)
Public	5 (2%)			Simple	84 (37%)
Patients	42 (19%)				
Caregivers	17 (7%)				
Physicians	193 (85%)				
Nurses	37 (16%)				
Other	23 (10%)				

^a^One trial could be categorized into several categories, and therefore, the sum of percentages may be over 100%

^b^Nine trials had multiple treatment arms and tested both simple and multicomponent interventions. Simple intervention was defined as having one intervention category with or without tailoring

Most commonly, studies were conducted in family medicine/general practice (*n* = 155, 68%), followed by internal medicine (*n* = 19, 8%), emergency medicine (*n* = 18, 8%), and pediatrics (*n* = 14, 6%) (Additional file 1, eFig. 2). The de-implementation intervention was targeted at physicians in 193 trials (85%). Most (*n* = 163, 72%) trials aimed to reduce use of drug treatments, typically antibiotics (*n* = 108, 48%). Besides reducing the use of practice, 42 trials (19%) additionally aimed to replace it with another practice. The most common (*n* = 145, 64%) rationale for de-implementation was “Evidence suggests more harms than benefits for the patient or community”, followed by “Evidence suggests little or no benefit from treatment or diagnostic test” (*n* = 115, 51%), and “Cost-effectiveness” (*n* = 70, 31%) (Table 2).

### Risk of bias

An allocation sequence was adequately generated in 224 of 227 studies (99%) and adequately concealed in 172 (76%). Blinding of data collection was adequate in 171 of 227 (75%) studies and of data analysts in 14 of 227 (6%). Out of 227 studies, 90 (40%) had little missing data, 33 (15%) had large missing data, and 104 (46%) did not report missing data. No or little baseline imbalance was found in 128 (56%) studies (Additional file 1, eFigs. 3 and 4).

### Study outcomes

The total volume of care was a reported study outcome in 194 (85%) studies, followed by low-value care use in 63 (28%), patient health outcomes in 58 (26%), and intention to reduce low-value care in 17 (7%) studies. In 34 trials (15%), authors reported changes in appropriate care, of which 16 studies reported an increase, 16 no effect, and 2 a decrease in appropriate care. In 186 studies (82%), authors reported at least some desired effect of the de-implementation intervention. Authors reported costs of the de-implementation interventions in 20 (9%) studies and the impact on healthcare costs in 45 (20%) studies.

### Conflicts of interest and funding

Authors reported having financial conflicts of interest (COI) in 33 studies (15%) and no financial COI in 124 (55%), while in 70 articles (31%), authors did not report information on financial COI. In 27 trials (12%), authors reported nonfinancial COI. Governments or universities funded 163 (72%), foundations 51 (22%), and private companies 16 (7%) studies; 8 (4%) studies reported no funding.

### Quality indicators

In cluster RCTs, the median number of clusters was 24 (IQR 44) (in trials published in 2010 or before 20 [IQR 31] and after 2010 30 [IQR 42]). Intra-cluster correlation (ICC) estimates were used to calculate sample size in 50 (34%) out of 145 cluster trials (in 28% until 2010 and 40% after 2010). The median follow-up time was 289 days (IQR 182) (273 days until 2010 and 335 days after 2010), while 16 (7%) trials gathered outcomes immediately after the intervention, and 9 trials did not report follow-up time (Additional file 1; eTable 1).

Out of 227 trials, 172 (76%; 71% of trials until 2010 and 81% after 2010) reported differences (in low-value care use) between baseline and after the intervention (follow-up) or provided prevalence estimates for baseline and after the intervention. Tailoring of the de-implementation intervention according to context was reported in 40 trials (18%; in 17% of trials until 2010 and 19% after 2010). The methods of tailoring included (i) surveys and focus groups with local professionals and patients (*n* = 21), (ii) identification of barriers for de-implementation and determinants of low-value care use (*n* = 20), (iii) local involvement in intervention planning (*n* = 8), and (iv) asking feedback from local professionals or/and patients (*n* = 4).

Of the 227 trials, 48 (21%; 19% of trials until 2010 and 23% after 2010) specified the theory or framework behind the de-implementation intervention (Additional file 1; eTable 2). Of these 48 trials, 25 used classic theories, 18 implementation theories, 8 evaluation frameworks, 2 determinant frameworks, and 1 process model (6 trials used 2 types of theories/frameworks). In trials with provider-level outcomes, 26 (12%; 12% of trials until 2010 and 11% after 2010) randomized on the patient level.

### Intervention categorization

**Fig. 2:**
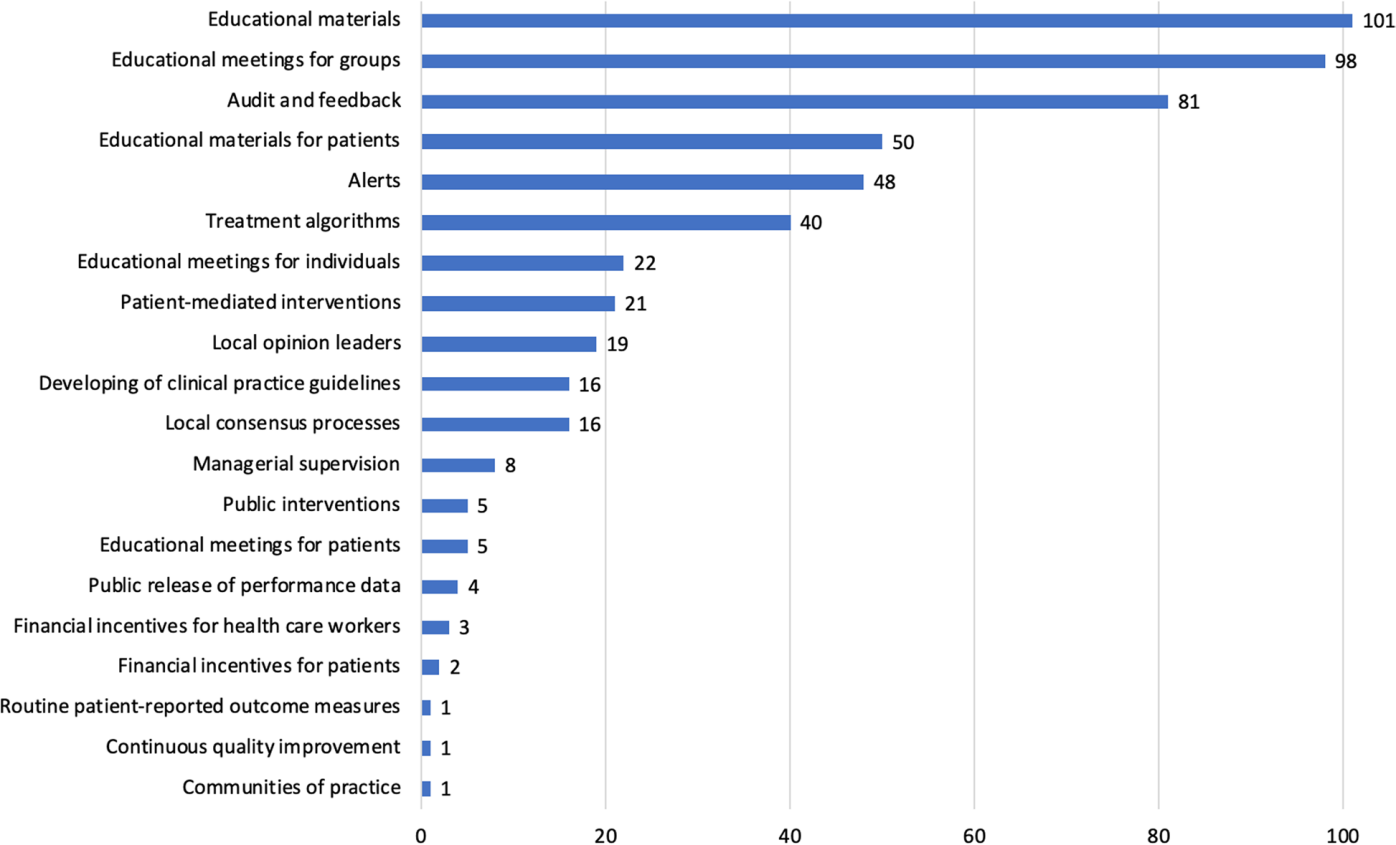
Number of randomized controlled trials in each intervention category

Most trials (*n* = 152, 67%) evaluated multicomponent interventions, that is, ones consisting of several components (Fig. 2). Educational materials (*n* = 101, 44%), educational meetings for groups (*n* = 98, 43%), and audit and feedback (*n* = 81, 36%) were the most studied intervention components. The most studied single-component interventions were alerts (*n* = 21, 25% of 84 trials testing simple interventions), followed by audit and feedback (*n* = 15, 18%), and educational meetings for healthcare worker groups (*n* = 12, 14%). A full description of the single-component interventions is presented in the Additional file 1 (eFig. 5).

## Discussion

We performed the first comprehensive systematic scoping review of de-implementation RCTs. We identified 227 RCTs, half published between 1982 and 2010 and the other half 2011–2021, indicating a substantial increase in research interest of de-implementation. Trials were typically conducted in primary care and tested educational interventions for physicians aiming to reduce use of drug treatments. We identified several study characteristics that may have led to unprecise effect estimates and limit applicability of the results in practice. These limitations include a small number of clusters in cluster randomized trials, potentially unreplicable study designs, and use of indirect, rather than low-value care-specific outcomes. To guide future research, we provided recommendations on how to address these issues (Table 3).

**Table 3:** Recommendations for planning de-implementation research

Problem	Explanation and elaboration	Recommendation	Evidence (identified in our scoping review)
**Complex interventions** Studying very complex interventions increases challenges in feasibility, replication, and evaluation of individual factors that affect the success of de-implementation	To progress the understanding of what works in de-implementation and making interventions more feasible, simpler interventions should be conducted. Simpler intervention means that there are fewer factors potentially affecting the success of de-implementation. When conducting simpler interventions, it is also easier to separate effective from ineffective factors. When conducting more complex interventions, process evaluation can improve the feasibility and help separate the important factors	Prefer simpler intervention designs	67% of studies had multiple intervention components, which usually leads to higher intervention complexity
**Human intervention deliverer** Generalizability decreases when the “human factor” (personal characteristics of the deliverers) affects the results of de-implementation	A human deliverer of the intervention may introduce confounding characteristics that affect the success of de-implementation. To improve the applicability of the results, studies should aim for higher number of intervention deliverers. When reporting the results, article should specify the number and characteristics of the deliverers used	Aim for larger number of intervention deliverers and describe the number and characteristics of the deliverers	50% of studies tested an intervention with educational sessions using a human intervention deliverer
**Small number of clusters** A small number of clusters decreases the reliability of effect estimates	The intra-cluster correlation coefficient is used to adjust sample sizes for between-cluster heterogeneity in treatment effects. This adjustment is often insufficient in small cluster randomized trials, as they produce imprecise estimates of heterogeneity, which may lead to unreliable effect estimates and false-positive results [21, 22]. Probability of false-positive results increases with higher between-cluster heterogeneity and smaller number of clusters (especially under 30 clusters) [21, 22]. Analyses may be corrected by small sample size correction methods, resulting in decreased statistical power. If the number of clusters is low, higher statistical power in individually randomized trials may outweigh the benefits acquired from cluster RCT design, avoiding contamination [23]	If the eligible number of clusters is low, consider performing an individually randomized trial. If number of clusters is small, consider using small sample size correction methods to decrease the risk of a false-positive result. Take the subsequent decrease in statistical power into account when calculating target sample size	In 145 cluster randomized trials, the median number of clusters was 24
**Dropouts** Dropouts of participants may lead to unreliable effect estimates	Trials should report dropouts for all intervention participants, including participants that were targeted with the de-implementation intervention and participants used as the measurement unit. Trials should separate between intervention participants that completely dropped out and who were replaced by new participants. To minimize dropouts, randomization should occur as close to the intervention as possible	Report dropouts for all intervention participants. Randomize as near to the start of the intervention as possible	Missing data led to a high risk of bias in 60% of studies, of which 76% were due to unreported data
**Heterogeneous study contexts** Diverse contextual factors may affect the outcome	Behavioral processes are usually tied to “local” context, including study environment and characteristics of the participants. These factors may impact participants’ behavior. Tailoring the intervention facilitates designing the intervention to target factors potentially important for the de-implementation. Examples include assessing barriers for change (and considering them in the intervention design) and including intervention targets in planning the intervention	Tailor the intervention to the study context	82% of the studies did not tailor the intervention to the study context
**Heterogeneous mechanisms of action** De-implementation interventions have diverse mechanisms of action	Theoretical knowledge helps to understand how and why de-implementation works. A theoretical background may not only increase chances of success but also improve the understanding of what works (and what does not work) in de-implementation. Examples include describing barriers and enablers for the de-implementation or describing who are involved and how they contribute to process of behavioral change	Use a theoretical background in the planning of the intervention	79%of the studies did not report a theoretical basis for the intervention
**Randomization unit** Randomization at a different level from the target level where the intervention primarily happens may result in loss of the randomization effect	Reducing the use of medical practices happens at the level of the medical provider. Therefore, if randomization happens at the level of the patient, the trial will not provide randomized data on provider-level outcomes. Even when the intervention target is the patient, the provider is usually involved in decision-making. Therefore, the intervention effect will occur on both provider and patient levels. Randomization is justified at the patient level when patient-level outcomes are measured or when the number of providers is large, representing several types of providers	Randomize at the same level as the intervention effect is measured	12% of the studies had provider-level outcome(s) but were randomized at the patient level
**Outcomes** Total volume of care outcomes may not represent changes in low-value care use	Total volume of care outcomes (including diagnosis-based outcomes) are vulnerable to bias, such as seasonal variability and diagnostic shifting [24]. Changes in these outcomes may not represent changes in actual low-value care use as the total volume of care includes both appropriate and inappropriate care. When measuring low-value care, comparing its use relative to the total volume of care or to appropriate care can help mitigate these biases	Use actual low-value care use outcomes whenever possible	28% of the studies measured actual low-value care use
**Cluster heterogeneity** Practice level variability in use of low-value care may be large	Baseline variability in low-value care use may be large [25]. As such, if the number of clusters is low, the baseline variability might lead to biased effect estimates	Compare low-value care use between the baseline and after the intervention	24% of the studies did not report baselines estimates or differences between the baseline and after the intervention

Our systematic scoping review identified several potential research gaps, including de-implementation in secondary and tertiary care settings, interventions targeted to other populations than physicians, diagnostic procedures, operative treatments, and de-implementation in non-Western societies. To fill these gaps, future RCTs could therefore investigate, for instance, de-implementation of preoperative testing in low-risk surgery [26, 27], operative treatment of low-risk disease [28, 29], and overuse of antibiotics in non-Western societies [30].

Earlier systematic and scoping reviews on de-implementation have focused on a narrow subject or included only a small number of RCTs (earlier systematic and scoping reviews listed in Additional file 1, eMethods 6). We included 227 de-implementation RCTs, which is substantially more than in previous reviews that included between 1 and 24 each. Indeed, we included 149 RCTs not included in any of the previous reviews.

Previous systematic reviews have suggested multicomponent interventions to be the most effective approach to de-implementation [4, 31]. Therefore, unsurprisingly, two-thirds of the identified 227 trials in our sample tested multicomponent interventions. The focus on often highly complex interventions has also, however, downsides. In addition to shortcomings in reporting of the interventions [32, 33], their complexity makes the repetition difficult. Context-specific intervention components and multifactorial intervention processes [34] increase the risk of missing important factors when replicating the intervention. Therefore, the value of conducting RCTs with interventions that are difficult to adapt to other settings may be limited. Conducting RCTs with simpler and more replicable interventions would be preferable [35–37].

Approximately, half of the 227 included RCTs tested educational session interventions. Educational interventions have been suggested to have modest benefits both in implementation and in de-implementation [31, 38, 39]. In addition, the applicability of the results of these RCTs may be limited due to “human factor” (Table 3). Instead of educational sessions, future educational studies could focus on more replicable interventions, for instance by integrating new information into decision-making pathways [37, 40, 41]. Furthermore, if a human deliverer is being used, having more deliverers and providing continuing educational support [42] in clinical work environments may increase the likelihood of efficiency (Table 3).

One of the main goals of our review was to guide future systematic reviews. Several methodological characteristics, or lack thereof, may lead to challenges in conducting these kinds of (systematic) reviews, including the following: (i) follow-up time and its measurement (some trials measure outcomes, such as practice use, during [24, 43] and others after [44, 45] the intervention), (ii) reporting of baseline data (some trials report practice use only after the intervention), (iii) variation in the intervention itself between individuals and studies (especially common when using complex interventions), and (iv) heterogeneity in study outcomes. To address these issues rising from study design heterogeneity, future systematic reviews could (i) explore the potential heterogeneity in de-implementation interventions, study contexts, and study designs when planning the analysis (for instance, by using logic models) [46, 47], (ii) rely on high-quality reporting standards to describe the study characteristics that may affect the analysis and replication/implementation of the included interventions [48], and (iii) assess the applicability of the studies [46, 47].

With increasing healthcare costs and limited resources, researchers and healthcare systems should focus on providing the best possible evidence on reducing the use of low-value care. Although we found increasing interest in de-implementation research, we also identified that many de-implementation RCTs use methods with high risk of bias. In general, low-quality methods increase research waste, and studies using such methods increase the risk of adapting ineffective de-implementation interventions. Failure to address these issues will emanate to patients, resulting in preventable harm and more use of low-value care.

### Limitations

Our systematic review has some limitations. First, although the search was designed to be as extensive as possible, we may have missed some relevant articles due to heterogenous indexing of de-implementation studies. On the other hand, we found 227 RCTs, of which 149 had not been identified by any of the earlier systematic reviews (Additional file 1, eMethods 6 and eTable 3). Second, same risk of bias criteria could not be used for individual and cluster RCTs. This may have led to unintended differences in individual and cluster RCT assessment. Third, interventions within categories of our refined taxonomy may still substantially vary. This may limit the adaptability of the taxonomy.

## Conclusions

This systematic scoping review identified 227 de-implementation RCTs, half published during the last decade and the other half during the three previous decades, indicating substantial increase in de-implementation research interest. We identified several areas with room for potential improvement, including more frequent use of simple intervention designs, more profound understanding and use of theoretical basis, and larger number of clusters in cluster trials. Addressing these issues would increase the trustworthiness of research results and replicability of interventions, leading to identification of useful de-implementation interventions and, ultimately, a decrease in the use of low-value practices.

## Supplementary Information

**Additional file 1: eFigure1.** Flow diagram. **eFigure 2.** Published studies per medical content area. **eFigure 3.** Risk of bias per question. **eFigure 4.** Risk of bias inside intervention categories. **eFigure 5.** Intervention components in single-component interventions. **eMethods 1.** Search strategies. **eMethods 2.** Risk of Bias Tool for RCTs of complex interventions. **eMethods 3.** Refined version of intervention taxonomy for de-implementation interventions. **eMethods 4.** Rationale for refined intervention taxonomy. **eMethods 5.** Rationale for outcome hierarchy of effectiveness outcomes in de-implementation. **eMethods 6.** Identified scoping and systematic reviews of de-implementation. **eTable 1.** Quality outcomes until 2010 and after. **eTable 2.** Theoretical background used in designing the interventions. **eTable 3.** Citations for included studies.

**Additional file 2.** Preferred Reporting Items for Systematic reviews and Meta-Analyses extension for Scoping Reviews (PRISMA-ScR) Checklist.

## Data Availability

The datasets generated during and/or analyzed during the current study are available from the corresponding author on reasonable request.

## Abbreviations

RCT: Randomized controlled trial
EPOC: Effective Practice and Organisation of Care
COI: Conflict of interest
