# Supplementary material for: Randomized controlled trials in de-implementation research: a systematic scoping review
Source: Implement Sci. 2022 Oct 1;17:65. doi: 10.1186/s13012-022-01238-z (PMC9526943; doi:10.1186/s13012-022-01238-z)
Supplement: Supplementary file 1 — Additional file 1: eFigure1. Flow diagram. eFigure 2. Published studies per medical content area. eFigure 3. Risk of bias per question. eFigure 4. Risk of bias inside intervention categories. eFigure 5. Intervention components in single-component interventions. eMethods 1. Search strategies. eMethods 2. Risk of Bias Tool for RCTs of complex interventions. eMethods 3. Refined version of intervention taxonomy for de-implementation interventions. eMethods 4. Rationale for refined intervention taxonomy. eMethods 5. Rationale for outcome hierarchy of effectiveness outcomes in de-implementation. eMethods 6. Identified scoping and systematic reviews of de-implementation. eTable 1. Quality outcomes until 2010 and after. eTable 2. Theoretical background used in designing the interventions. eTable 3. Citations for included studies. [file 13012_2022_1238_MOESM1_ESM.docx]

# Additional file 1

**Table of Contents**  Page

1. **eFigure 1.** Flow diagram 2
2. **eMethods 1.** Search strategies 3
   1. Search strategy in Scopus
   2. Search strategy in Ovid MEDLINE
3. **eMethods 2.** Risk of Bias Tool for RCTs of complex interventions 6
4. **eMethods 3.** Refined version of intervention taxonomy for de-implementation interventions 11
5. **eMethods 4.** Rationale for refined intervention taxonomy 13
6. **eMethods 5.** Rationale for outcome hierarchy of effectiveness outcomes in de-implementation 15
7. **eMethods 6.** Identified scoping and systematic reviews of de-implementation 16
8. **eFigure 2.** Published studies per medical content area 18
9. **eFigure 3**. Risk of bias per question 19
10. **eFigure 4.** Risk of bias inside intervention categories 20
11. **eFigure 5.** Intervention components in studies with one component 21
12. **eTable 1.** Quality outcomes until 2010 and after 22
13. **eTable 2.** Theoretical background used in designing the interventions 23
14. **eTable 3.** Citations for included studies 28

**eFigure1.** Flow diagram

**Identification of studies via databases and registers**

Records removed *before screening*:

Duplicate records removed (n = 2082)

Records identified from*:

Databases (n = 12815)

Scopus (n = 7489)

Medline (n = 5326)

**Identification**

Records screened

(n = 10733)

Records excluded

(n = 9708)

Reports sought for retrieval

(n = 1025)

Reports not retrieved

(n = 2)

**Screening**

Reports excluded:

Not an RCT (n = 139)

Not testing de-implementation (n = 614)

Not testing intervention to reduce use of clinical practice (n = 5)

No outcomes of interest (n = 1)

Reports assessed for eligibility

(n = 1023)

Studies included in review

(n = 227)

Reports of included studies

(n = 240)

36 eligible articles idetified from systematic reviews(n=62), protocols and post-hoc analysis

**Included**

*Consider, if feasible to do so, reporting the number of records identified from each database or register searched (rather than the total number across all databases/registers).

*From:*  Page MJ, McKenzie JE, Bossuyt PM, Boutron I, Hoffmann TC, Mulrow CD, et al. The PRISMA 2020 statement: an updated guideline for reporting systematic reviews. BMJ 2021;372:n71. doi: 10.1136/bmj.n71

For more information, visit: <http://www.prisma-statement.org/>

**eMethods 1.** Search strategies

**Scopus**

Search history 26.5.2021

( ( TITLE-ABS-KEY ( "randomized controlled trial" ) ) OR ( TITLE-ABS-KEY ( "controlled clinical trial" ) ) OR ( ABS ( randomized OR placebo OR randomly ) ) OR ( TITLE ( trial ) ) ) AND ( ( ( TITLE-ABS-KEY ( abandon* OR de-list* OR dis-invest* OR discontinu* OR dis-continu* OR decommiss* OR de-commiss* OR deadopt* OR de-adopt* OR de-implement* OR deimplement* OR reduc* OR remov* OR stop* OR relinquish* OR disadopt* OR disinvest* OR de-prescri* OR deprescri* ) ) OR ( TITLE-ABS-KEY ( ( decreas* W/3 "use" ) OR ( declin* W/3 "use" ) OR ( drop* W/3 "use" ) OR ( decreas* W/3 rate* ) ) ) OR ( TITLE-ABS-KEY ( withdraw* OR replac* OR reallocat* OR re-allocat* ) ) OR ( TITLE-ABS-KEY ( "change* in use" OR "change* in practice*" ) ) OR ( TITLE-ABS-KEY ( improv* W/4 "use" ) ) OR ( TITLE-ABS-KEY ( reduc* W/4 "use" ) ) OR ( TITLE-ABS-KEY ( change* W/4 "use" OR change* W/3 practice* ) ) ) AND ( ( TITLE-ABS-KEY ( inappropriate W/3 prescri* ) ) OR ( TITLE-ABS-KEY ( "Health Service*" W/1 misus* ) ) OR ( TITLE-ABS-KEY ( low-value ) ) OR ( TITLE-ABS-KEY ( ( overutili* OR overus* OR overdiagnos* OR overtreat* OR overmedicat* OR overprescrib* ) ) ) OR ( TITLE-ABS-KEY ( ( unnecessary OR ineffective OR useless OR inefficient OR valueless ) W/1 ( care OR usage OR utilisation OR utilization OR treatment* OR intervention* OR practice* OR procedure OR drug* OR therap* OR technolog* OR device* OR surg* OR test* OR lab* OR imaging ) ) ) OR ( TITLE-ABS-KEY ( "unnecessary use" ) ) OR ( TITLE-ABS-KEY ( "INAPPROPRIATE use" ) ) OR ( TITLE-ABS-KEY ( obsolete ) ) OR ( TITLE-ABS-KEY ( contradict* OR refute* OR reassess* OR re-assess* OR re-apprais* OR reapprais* OR revers ) ) OR ( TITLE-ABS-KEY ( "Guideline Adherence" ) ) ) )

( ( TITLE-ABS ( "randomized controlled trial" ) ) OR ( TITLE-ABS ( "controlled clinical trial" ) ) OR ( TITLE-ABS ( randomized ) ) OR ( TITLE-ABS ( placebo ) ) OR ( TITLE-ABS ( randomly ) ) OR ( TITLE ( trial ) ) ) AND ( ( TITLE-ABS-KEY ( "Unnecessary Procedure*" ) ) OR ( TITLE-ABS-KEY ( "prescription rate*" ) ) OR ( TITLE-ABS-KEY ( "Guideline adherence" ) ) OR ( TITLE-ABS-KEY ( "too much medicine" ) ) OR ( TITLE-ABS-KEY ( "choosing wisely" ) ) OR ( TITLE-ABS-KEY ( "do not do" ) ) OR ( TITLE-ABS-KEY ( deprescription* ) ) OR ( TITLE-ABS-KEY ( guideline* W/3 implementation* ) ) OR ( TITLE-ABS-KEY ( reduc* W/4 prescri* ) ) OR ( TITLE-ABS-KEY ( unnecessary W/4 "use" ) ) OR ( TITLE-ABS-KEY ( unnecessary W/4 prescri* ) ) OR ( TITLE-ABS-KEY ( "Inappropriate prescri*" ) ) OR ( TITLE-ABS-KEY ( decreas* W/3 referrals ) )

**Medline**

Database: Ovid MEDLINE(R) ALL <1946 to May 24, 2021>

Search Strategy:

--------------------------------------------------------------------------------

1 Unnecessary Procedures/

2 prescription rate*.tw.

3 Guideline adherence.tw.

4 too much medicine.tw.

5 choosing wisely.tw.

6 "do not do".tw.

7 deprescriptions/

8 (guideline* adj3 implementation*).tw.

9 Practice Patterns, Physicians'/sn, st

10 Physicians, Family/st, sn [Standards, Statistics & Numerical Data]

11 exp Drug Utilization/sn [Statistics & Numerical Data]

12 randomized controlled trial.pt.

13 controlled clinical trial.pt.

14 randomized.ab.

15 placebo.ab.

16 clinical trials as topic.sh.

17 randomly.ab.

18 trial.ti.

19 12 or 13 or 14 or 15 or 16 or 17 or 18

20 exp animals/ not humans.sh.

21 19 not 20

22 1 or 2 or 3 or 4 or 5 or 6 or 7 or 9 or 10 or 11

23 21 and 22

24 (reduc* adj4 prescri*).tw.

25 (unnecessary adj4 "use").mp. [mp=title, abstract, original title, name of substance word, subject heading word, floating sub-heading word, keyword heading word, organism supplementary concept word, protocol supplementary concept word, rare disease supplementary concept word, unique identifier, synonyms]

26 (unnecessary adj4 prescri*).tw.

27 Inappropriate Prescribing/

28 (decreas* adj3 referrals).tw.

29 inappropriate prescri*.tw.

30 24 or 25 or 26 or 27 or 28 or 29

31 21 and 30

32 23 or 31

Database: Ovid MEDLINE(R) ALL <1946 to May 24, 2021>

Search Strategy:

--------------------------------------------------------------------------------

1 exp Health Services Misuse/

2 Inappropriate Prescribing/

3 low-value.tw.

4 (overutili* or overus* or overdiagnos* or overtreat* or overmedicat* or overprescrib*).tw.

5 ((unnecessary or ineffective or useless or inefficient or valueless) adj (care or usage or utilisation or utilization or treatment* or intervention* or practice* or procedure or drug* or therap* or technolog* or device* or surg* or test* or lab* or imaging)).tw.

6 "unnecessary use".tw.

7 Inappropriate Prescri*.tw.

8 "INAPPROPRIATE use".tw.

9 obsolete.tw.

10 (contradict* or refute* or reassess* or re-assess* or re-apprais* or reapprais* or revers).tw.

11 exp Guideline Adherence/

12 1 or 2 or 3 or 4 or 5 or 6 or 7 or 8 or 9

13 (abandon* or de-list* or dis-invest* or discontinu* or dis-continu* or decommiss* or de-commiss* or deadopt* or de-adopt* or de-implement* or deimplement* or reduc* or remov* or stop* or relinquish* or disadopt* or DISINVEST* or de-prescri* or Deprescri*).tw.

14 ((decreas* adj3 "use") or (declin* adj3 "use") or (drop* adj3 "use") or (decreas* adj3 rate*)).mp.

15 (withdraw* or replac* or reallocat* or re-allocat*).tw.

16 ("change* in use" or "change* in practice*").tw.

17 (improv* adj4 "use").mp.

18 ((change adj4 "use") or "change* in practice").mp.

19 (reduc* adj4 "use").tw.

20 13 or 14 or 16 or 17 or 18 or 19

21 12 and 20

22 randomized controlled trial.pt.

23 controlled clinical trial.pt.

24 randomized.ab.

25 placebo.ab.

26 clinical trials as topic.sh.

27 randomly.ab.

28 trial.ti.

29 22 or 23 or 24 or 25 or 26 or 27 or 28

30 exp animals/ not humans.sh.

31 29 not 30

32 21 and 31

**eMethods 2.** Risk of Bias Tool for RCTs of complex interventions

1. **Randomization/imbalance of prognostic factors**
   1. **Was the allocation sequence adequately generated?**

Definitely yes Probably yes Probably no Definitely no

(low risk of bias) (high risk of bias)

The use of a random component should be sufficient for adequate sequence generation. This could be achieved by allocating interventions using methods such as repeated coin-tossing, throwing dice or dealing previously shuffled cards. If the allocation was by telephone or Internet, the randomization was done through a computer system.

Examples of low risk of bias (“definitely yes”): Referring to a random number table; Using a computer random number generator; Coin tossing; Shuffling cards or envelopes; Throwing dice; Drawing of lots; Minimization with or without a random element.

Examples of high risk of bias (“definitely no”): Sequence generated by odd or even date of birth; Sequence generated by some rule based on date (or day) of admission; Sequence generated by some rule based on hospital or clinic record number; Allocation by judgement of the clinician; Allocation by preference of the participant; Allocation based on the results of a laboratory test or a series of tests; Allocation by availability of the intervention.

If they say “randomized” and give no more information regarding sequence generation, the process was probably low risk of bias, so, answer “Probably yes”.

- 1. **Was the allocation adequately concealed?**

Definitely yes Probably yes Probably no Definitely no

(low risk of bias) (high risk of bias)

In randomized trials, allocation concealment strategies hide the method of sorting trial participants into treatment groups so that this knowledge cannot be exploited. Adequate allocation concealment serves to prevent trial investigators/recruiters from choosing treatment allocations for individuals/patients. Studies with poor allocation concealment (or none at all) are prone to selection bias. Trials where participants are recruited before randomization are low risk of bias. Trials where participants are recruited between randomization and the beginning of the intervention are usually high risk of bias.

If there are multiple levels of recruitment, consider allocation concealment on the lowest level. For instance, educational intervention targeted to physicians: clusters are health care centers, physicians are participants to whom the educational intervention was targeted and patients were participants to whom the medical intervention was targeted. If patients are recruited after the randomisation we will consider the study as high risk of bias. An exception is, however, if all patients from the physician were analysed or otherwise it was impossible for the physician to decide which patients were included. In this case we will consider study as low risk of bias.

Examples of low risk of bias (“definitely yes”):

1. i) the unit of allocation was by patient or episode of care AND ii) there was some form of centralized randomization scheme and on-site computer system OR sealed opaque envelopes were used.
2. i) the unit of allocation was by institution, team or professional AND ii) less than 5% were recruited (at the lowest level) after the randomization
3. i) the unit of allocation was by institution, team or professional AND ii) Lowest level of recruitment was conducted after the randomization AND iii) recruiter(s) were blinded to study groups OR all participants were recruited

Examples of low risk of bias (“probably yes”):

1. i) the unit of allocation was by institution, team or professional AND ii) less than 10% were recruited (at the lowest level) after the randomization

Examples of high risk of bias (“probably no”):

1. the unit of allocation was by patient or episode of care and there was some form of centralized randomization scheme and allocation concealment was not reported.
2. the unit of allocation was by institution, team or professional and recruitment of study participants and It’s unclear how many participants were recruited after the randomization.

Examples of high risk of bias (“definitely no”):

1. the unit of allocation was by patient or episode of care AND Using an open random allocation schedule (e.g. a list of random numbers) OR Assignment envelopes were used without appropriate safeguards (e.g. if envelopes were unsealed or non-opaque or not sequentially numbered) OR Alternation or rotation OR Date of birth OR Case record number OR Any other explicitly unconcealed procedure.
2. i) the unit of allocation was by institution, team or professional AND ii) allocation was not performed for all at the start of the study (over 10% recruitment of study participants after the randomization)

1. **Blinding. Was knowledge of the allocated interventions adequately prevented?**
   1. **Were data collectors/outcome assessors blinded?**

Definitely yes Probably yes Probably no Definitely no

(low risk of bias) (high risk of bias)

Low risk of bias (“definitely yes”):

Data were collected from medical records or other database and blinding of data collectors was reported.

In other situations, if data collectors did not know to which group study participants belong.

Low risk of bias (“probably yes”):

Data were collected from medical records or other database and blinding of data collectors was not reported.

High risk of bias (“probably no”):

In trials where data were not collected from medical records or other database, if blinding was not reported.

High risk of bias (“definitely no”):

Data were collected from medical records or other database and it was somehow stated that data collectors were not blinded or non-blinding is obvious other way.

In other situations, if non-blinding was reported.

If there were several ways of collecting the data, assess the way that primary outcome(s) was collected. If there was no reported primary outcome(s), primarily assess regarding the outcome of prevalence of low-value care/total volume of care was collected and secondarily assess regarding the outcome of intention/perception to reduce low-value care.

- 1. **Were data analysts blinded?**

Definitely yes Probably yes Probably no Definitely no

(low risk of bias) (high risk of bias)

Answer “Probably no” unless there is some specific indication implying that data analysts were blinded.

1. **Missing data (by primary outcome):**

Definitely yes Probably yes Probably no Definitely no

(high risk of bias) (low risk of bias)

Thresholds for risk of bias judgements. Respond:

“Definitely no”:

Less than 5% of the primary outcome data is missing (low risk of bias)

“Probably no”:

At least 5% but less than 10% of the outcome data is missing (low risk of bias)

“Probably yes”:

At least 10% but less than 20% of the outcome data is missing (high risk of bias)

“Definitely yes”:

At least 20% of the outcome data is missing (high risk of Bias)

Preferably use the primary outcome for assessment of missing data/drop outs. If there is no primary outcome, i) primarily use the outcome(s) measuring total volume of care/prevalence of low-value care; and ii) secondarily outcome(s) measuring intention/perception/willingness to reduce low-value care.

If there is one primary outcomes, use the proportion of missing data for judgment. If there are several primary outcomes (or if there is no primary outcome and other outcome types are used), use median values of missing outcome data for outcome category.

When judging missing data, please, remember to consider all levels of recruitment where missing data/drop outs are possible. If drop outs are highly unlikely in every level, answer “probably no” (in individually randomised trials this means only one level).

Drop outs are considered as highly unlikely when recruitment/allocation, intervention and outcome measurement happens within the same encounter.

For example, **i)** patient may be recruited, allocated, given the intervention and outcome assessed/measured within the same visit to the health care center or **ii)** physician may be recruited, allocated, intervention given and outcome measured within the same encounter (e.g. authors use survey to measure willingness to reduce use of low-value care after educational intervention and everything from allocation to measurement is done within the same encounter). In these situations, record as “probably no”.

Drop outs that are included in the analysis (intention to treat principle) are not considered as missing outcome data. Sometimes authors use “intention to treat principle” in the analysis (and include participants that did drop out but could still be followed up to measure the outcomes), but there is still drop outs with missing outcome data that they can’t include in the analysis.

1. **Were there baseline imbalances at the start of the trial?**

Definitely yes Probably yes Probably no Definitely no

(high risk of bias) (low risk of bias)

Consider baseline estimates of (primary) outcome and age/work experience between intervention and control groups.

Thresholds for risk of bias judgements. Respond:

“definitely no”: Relative difference (compared to control group) is less than 5% (low risk of bias)

“probably no”: Relative difference (compared to control group) at least 5% but less than 10%

“probably yes”: Relative difference (compared to control group) at least 10% but less than 20%

“definitely yes”: Relative difference (compared to control group) at least 20% (high risk of bias)

Examples of relative difference:

if use of antibiotics 80% in intervention and 60% in control group relative difference here is not 20% but it is 33%

if median age is 68 years in intervention and 60 years in control group, relative difference is here 13%

If not reported, answer “probably yes”. An exception is, however, when cluster unit is team or institution AND the number of clusters is over 20 OR if trial is individually randomized/cluster unit individual professional AND number of clusters/individuals is over 100. In this case answer “probably no”.

If there are more than 1 primary outcome, we will use the median value (of proportion of missing data). If no outcome is clearly reported as primary outcome, we will use the median value (of proportion of missing data) of all outcomes that measure prevalence of low value care use. If there are no outcomes reported measuring prevalence of low value care use, we will use the median value (of proportion of missing data) of outcomes that measure intention/perception to reduce low-value care.

1. **Other risk of bias?**
   1. **choose at least one**
      1. **Contamination**
      2. **Selective reporting**
      3. **Other**
      4. **No**

**eMethods 3.** Refined version of intervention taxonomy for de-implementation interventions

*With the refined de-implementation intervention taxonomy, we aim to provide categorization in a way that de-implementation interventions are comparable and comprehensively show the spectrum of intervention types that have been studied. To achieve this, we 1) eliminated unnecessary overlaps between intervention categories, 2) separated interventions to several categories if there were clear justifications to do so and 3) made clarifications to existing categories if they were not suitable to describe current de-implementation literature.*

**Audit and feedback**

A summary of health workers’ performance over a period of time, given to them in a written, electronic or verbal format. The summary may include recommendations for clinical action or comparing individual/unit performance to peers.

**Alerts**

Manual or computerized system that alerts healthcare personnel when specific criteria in patients’ care, treatment or clinical outlook fulfill. Including added information in electronic health record systems that is meant to prompt health care workers to certain decisions.

**Treatment algorithm**

Manual or computerized treatment algorithm that helps health care workers with diagnosis and treatment choices.

**Communities of practice**

Groups of people with a common interest who deepen their knowledge and expertise in this area by interacting on an ongoing basis.

**Continuous quality improvement**

An iterative process to review and improve care that includes involvement of healthcare teams, analysis of a process or system, a structured process improvement method or problem-solving approach, and use of data analysis to assess changes.

**Educational materials**

Distribution to individuals, or groups, of educational materials to support clinical care, i.e., any intervention in which knowledge is distributed. For example, this may be facilitated by the internet, learning critical appraisal skills; skills for electronic retrieval of information, diagnostic formulation; question formulation.

**Educational meetings for individuals**

All educational meeting targeted on individuals. Including face-to-face visits and phone calls.

**Educational meetings for groups**

All educational meetings targeted on groups. Including online and face-to-face meetings, courses, workshops, conferences or other educational meetings, outreach visits and academic detailing. In educational meetings, participants have possibility to interact with the educator.

**Developing of Clinical practice guidelines**

Clinical guidelines are systematically developed statements to assist healthcare providers and patients to decide on appropriate health care for specific clinical circumstances'(US IOM).

**Local consensus process**

Formal or informal local consensus processes, for example agreeing a clinical protocol to manage a patient group, adapting a guideline for a local health system or promoting the implementation of guidelines. Including local development of guidelines, care protocols, decision supports and making commitment to follow guidelines/other clinical decision rules.

**Local opinion leaders**

The identification and use of identifiable local opinion leaders to promote good clinical practice.

**Managerial supervision**

Supervision visits by health staff. Including consultation.

**Patient-mediated interventions**

Any intervention aimed at changing the performance of healthcare professionals through interactions with patients, or information provided by patients. Including Shared decision making.

**Public release of performance data**

Informing the public about healthcare providers by the release of performance data in written or electronic form.

**Routine patient-reported outcome measures**

Routine administration and reporting of patient-reported outcome measures to providers and/or patients.

**Financial incentives for health care workers**

Money or financial benefit given to health care workers to prompt certain action.

**Financial incentives for patients**

Money or other financial benefit given to patients to prompt certain action.

**Tailored interventions**

Context has been considered in planning and selection of the interventions used. Including assessment of barriers to change and including participants from the intervention target group in planning the intervention.

**Educational meetings for patients**

Individual or group educational sessions for patients. In educational meetings, participants have possibility to interact with the educator.

**Educational material for patients**

Educational material distributed/shown to patients. Including web-based and paper-based material.

**Public interventions**

Interventions targeted to public. Including TV or radio campaigns.

**eMethods 4.** Rationale for refined intervention taxonomy

The EPOC taxonomy of health systems interventions have lastly been revised in 2015. The research area of de-implementation has evolved a lot in recent years and the revised taxonomy does not represent well the current de-implementation research literature. All interventions can’t be categorized with the current taxonomy and some interventions unnecessarily overlap in several categories.

**Aim**

With the refined de-implementation intervention taxonomy, we aim to provide categorization in a way that de-implementation interventions are comparable and comprehensively show the spectrum of intervention types that have been studied. To achieve this we 1) eliminated unnecessary overlaps between intervention categories, 2) separated interventions to several categories if there were clear justifications to do so and 3)made clarifications to existing categories if they was not suitable to describe current de-implementation literature.

**Combined categories**

The EPOC taxonomy had 4 categories for educational session interventions for health care staff: educational games, educational meetings, educational outreach visits (or academic detailing), interprofessional education. Although, educational games could also include other types of interventions than educational sessions, it is mostly included in educational sessions. After going assessing intervention types in our sample, major proportion of educational interventions (and components of interventions) could be categorized into both educational meetings and educational outreach visits. We also did not find any reason why specifically educational games or interprofessional education should be the intervention types that are separated from broader group of educational interventions. Educational interventions are very heterogenous group of interventions and separate taxonomy for educational implementation/de-implementation interventions would be justified.

Considering reasons above, we decided to divide educational session interventions in 2 groups: one-on-one educational meetings and group educational meetings. This categorization was also used by Mazza et al. in their guideline implementation taxonomy (1)

Reminders category were also very heterogenous group. It could be separated into 2 different groups: decision supports and alerts(reminders). Interventions in which decision support are once distributed differ fundamentally from intervention types where systematic alerts prompt health care workers to certain decision when prespecified clinical incidents emerge. “clinical incident reporting” was included in alert category. It was rarely used and we considered the idea to be very close to “alert” type of interventions.

“Monitoring the performance of the delivery of healthcare” was combined with audit and feedback because it unnecessarily overlaps with “audit and feedback” category and was not used as separate category in our sample. This could be more useful with more specified definition, but it was not relevant in our sample.

**Updated Definitions**

Definition of “patient-mediated interventions” we updated to only include interventions were information were provided by patients or interactions with patients were promoted.

We updated “managerial supervision” to include any type of consultation interventions.

We updated “Local consensus process” definition by adding “Including local development of guidelines, care protocols, decision supports and making commitment to follow guidelines/other clinical decision rules.”

We updated “tailored interventions” definition to “Intervention context has been considered in planning and selection of the interventions used. Including assessment of barriers to change and including participants from the intervention target group in planning the intervention”. Previous definition definition only included interventions where barriers for change was assessed. We did not consider this to be the only way of considering the context for the intervention and therefore the definition is now broader.

**New categories**

We added 2 new categories for interventions where educational content were provider to patients: educational meetings for patients and educational material for patients.

We added 2 new categories for financial incentives: financial incentives for health care professionals and financial incentives for patients.

**Limitations**

Our categorization represents the spectrum and types of the interventions in de-implementation literature, but it is not well suited to detailed analysis on what works in de-implementations as most categories include heterogenous types of interventions inside them.

1. Mazza D, Bairstow P, Buchan H, et al. Refining a taxonomy for guideline implementation: results of an exercise in abstract classification. *Implement Sci*. 2013;8:32. Published 2013 Mar 15. doi:10.1186/1748-5908-8-32

**eMethods 5.** Rationale for outcome hierarchy of effectiveness outcomes in de-implementation

We developed outcome hierarchy for effectiveness outcomes in de-implementation research. There is no standard way of doing the categorization. We used Kirkpatrick levels in the development (1). There is one categorization for de-implementation outcomes by Prusaczyk et al. (which divides outcomes to 8 categories: acceptability, adoption, appropriateness, cost, feasibility, fidelity, penetration, sustainability), but does not categorize effectiveness outcomes used in de-implementation literature (2).

Kirkpatrick levels have been developed for educational interventions. As de-implementation interventions also include other types of interventions, we modified it to fit the whole de-implementation literature and to concentrate in effectiveness of the interventions.

We excluded category for modifications of knowledge/skills as it was not related to all types of de-implementation interventions and it is not often measured in RCT setting. Categories 2a and 3 were combined into one category (intention/perception to reduce use of clinical practice).

1. Yardley S, Dornan T. Kirkpatrick's levels and education 'evidence'. Med Educ. 2012 Jan;46(1):97-106. doi: 10.1111/j.1365-2923.2011.04076.x. PMID: 22150201.
2. Prusaczyk, B., Swindle, T. & Curran, G. Defining and conceptualizing outcomes for de-implementation: key distinctions from implementation outcomes. *Implement Sci Commun* **1,**43 (2020). <https://doi.org/10.1186/s43058-020-00035-3>

**eMethods 6.** Identified scoping and systematic reviews of de-implementation

| Chaudhuri D, Montgomery A, Gulenchyn K, Mitchell M, Joseph P. Effectiveness of Quality Improvement Interventions at Reducing Inappropriate Cardiac Imaging: A Systematic Review and Meta-Analysis. *Circ Cardiovasc Qual Outcomes*. 2016;9(1):7-13. doi:10.1161/CIRCOUTCOMES.115.001836 |
| --- |
| Cliff BQ, Avanceña ALV, Hirth RA, Lee SD. The Impact of Choosing Wisely Interventions on Low-Value Medical Services: A Systematic Review. Milbank Q. 2021;99(4):1024-1058. doi:10.1111/1468-0009.12531 |
| Clyne B, Fitzgerald C, Quinlan A, et al. Interventions to Address Potentially Inappropriate Prescribing in Community-Dwelling Older Adults: A Systematic Review of Randomized Controlled Trials. *J Am Geriatr Soc*. 2016;64(6):1210-1222. doi:10.1111/jgs.14133 |
| Colla CH, Mainor AJ, Hargreaves C, Sequist T, Morden N. Interventions Aimed at Reducing Use of Low-Value Health Services: A Systematic Review. Med Care Res Rev. 2017;74(5):507-550. doi:10.1177/1077558716656970 |
| de Bont EG, Alink M, Falkenberg FC, Dinant GJ, Cals JW. Patient information leaflets to reduce antibiotic use and reconsultation rates in general practice: a systematic review. *BMJ Open*. 2015;5(6):e007612. Published 2015 Jun 3. doi:10.1136/bmjopen-2015-007612 |
| Desai S, Liu C, Kirkland SW, Krebs LD, Keto-Lambert D, Rowe BH. Effectiveness of Implementing Evidence-based Interventions to Reduce C-spine Image Ordering in the Emergency Department: A Systematic Review. *Acad Emerg Med*. 2018;25(6):672-683. doi:10.1111/acem.13364 |
| Fleming A, Browne J, Byrne S. The effect of interventions to reduce potentially inappropriate antibiotic prescribing in long-term care facilities: a systematic review of randomised controlled trials. *Drugs Aging*. 2013;30(6):401-408. doi:10.1007/s40266-013-0066-z |
| Chen I, Opiyo N, Tavender E, et al. Non-clinical interventions for reducing unnecessary caesarean section. *Cochrane Database Syst Rev*. 2018;9(9):CD005528. Published 2018 Sep 28. doi:10.1002/14651858.CD005528.pub3 |
| Hiscock H, Neely RJ, Warren H, Soon J, Georgiou A. Reducing Unnecessary Imaging and Pathology Tests: A Systematic Review. *Pediatrics*. 2018;141(2):e20172862. doi:10.1542/peds.2017-2862 |
| Hu Y, Walley J, Chou R, et al. Interventions to reduce childhood antibiotic prescribing for upper respiratory infections: systematic review and meta-analysis. *J Epidemiol Community Health*. 2016;70(12):1162-1170. doi:10.1136/jech-2015-206543 |
| Jenkins HJ, Hancock MJ, French SD, Maher CG, Engel RM, Magnussen JS. Effectiveness of interventions designed to reduce the use of imaging for low-back pain: a systematic review. *CMAJ*. 2015;187(6):401-408. doi:10.1503/cmaj.141183 |
| Köchling A, Löffler C, Reinsch S, et al. Reduction of antibiotic prescriptions for acute respiratory tract infections in primary care: a systematic review. *Implement Sci*. 2018;13(1):47. Published 2018 Mar 20. doi:10.1186/s13012-018-0732-y |
| Mokhar A, Topp J, Härter M, et al. Patient-centered care interventions to reduce the inappropriate prescription and use of benzodiazepines and z-drugs: a systematic review. *PeerJ*. 2018;6:e5535. Published 2018 Oct 8. doi:10.7717/peerj.5535 |
| Mortazhejri, S., Hong, P.J., Yu, A.M. *et al.* Systematic review of patient-oriented interventions to reduce unnecessary use of antibiotics for upper respiratory tract infections. *Syst Rev* **9,**106 (2020). https://doi.org/10.1186/s13643-020-01359-w |
| Ranji SR, Steinman MA, Shojania KG, Gonzales R. Interventions to reduce unnecessary antibiotic prescribing: a systematic review and quantitative analysis. *Med Care*. 2008;46(8):847-862. doi:10.1097/MLR.0b013e318178eabd |
| Rietbergen, T., Spoon, D., Brunsveld-Reinders, A.H. *et al.* Effects of de-implementation strategies aimed at reducing low-value nursing procedures: a systematic review and meta-analysis. *Implementation Sci* **15,**38 (2020). https://doi.org/10.1186/s13012-020-00995-z |
| Santos NSD, Marengo LL, Moraes FDS, Barberato Filho S. Interventions to reduce the prescription of inappropriate medicines in older patients. *Rev Saude Publica*. 2019;53:7. Published 2019 Jan 31. doi:10.11606/S1518-8787.2019053000781 |
| Sypes EE, de Grood C, Whalen-Browne L, et al. Engaging patients in de-implementation interventions to reduce low-value clinical care: a systematic review and meta-analysis. *BMC Med*. 2020;18(1):116. Published 2020 May 8. doi:10.1186/s12916-020-01567-0 |
| Tonkin-Crine SK, Tan PS, van Hecke O, et al. Clinician-targeted interventions to influence antibiotic prescribing behaviour for acute respiratory infections in primary care: an overview of systematic reviews. *Cochrane Database Syst Rev*. 2017;9(9):CD012252. Published 2017 Sep 7. doi:10.1002/14651858.CD012252.pub2 |
| Vodicka TA, Thompson M, Lucas P, et al. Reducing antibiotic prescribing for children with respiratory tract infections in primary care: a systematic review. *Br J Gen Pract*. 2013;63(612):e445-e454. doi:10.3399/bjgp13X669167 |
| Xiong Z, Chen H. Interventions to reduce unnecessary central venous catheter use to prevent central-line-associated bloodstream infections in adults: A systematic review. *Infect Control Hosp Epidemiol*. 2018;39(12):1442-1448. doi:10.1017/ice.2018.250 |
| Zhelev Z, Abbott R, Rogers M, et al. Effectiveness of interventions to reduce ordering of thyroid function tests: a systematic review. *BMJ Open*. 2016;6(6):e010065. Published 2016 Jun 3. doi:10.1136/bmjopen-2015-010065 |

**eFigure 2.** Published studies per medical content area


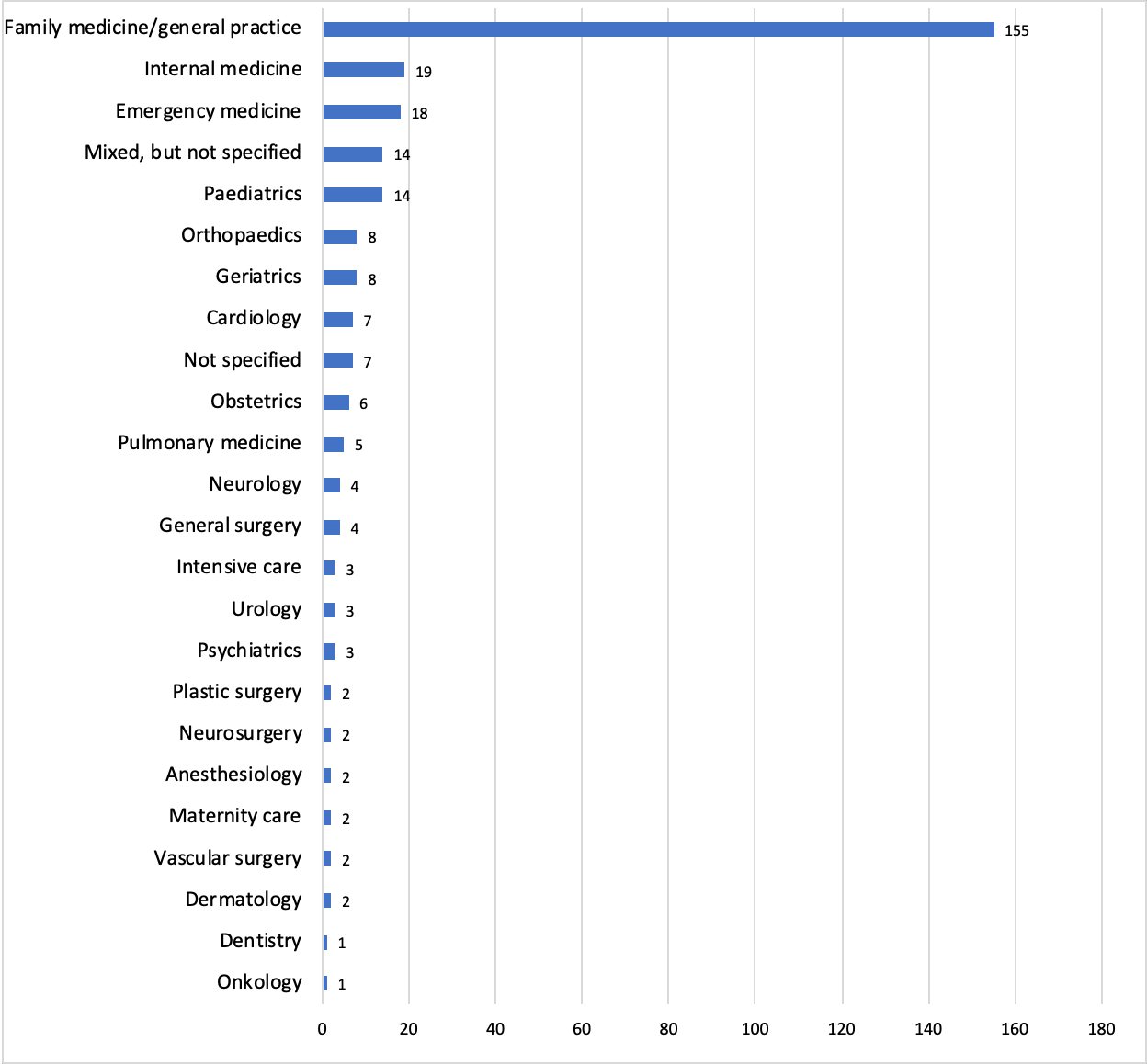


**eFigure 3**. Risk of bias per question


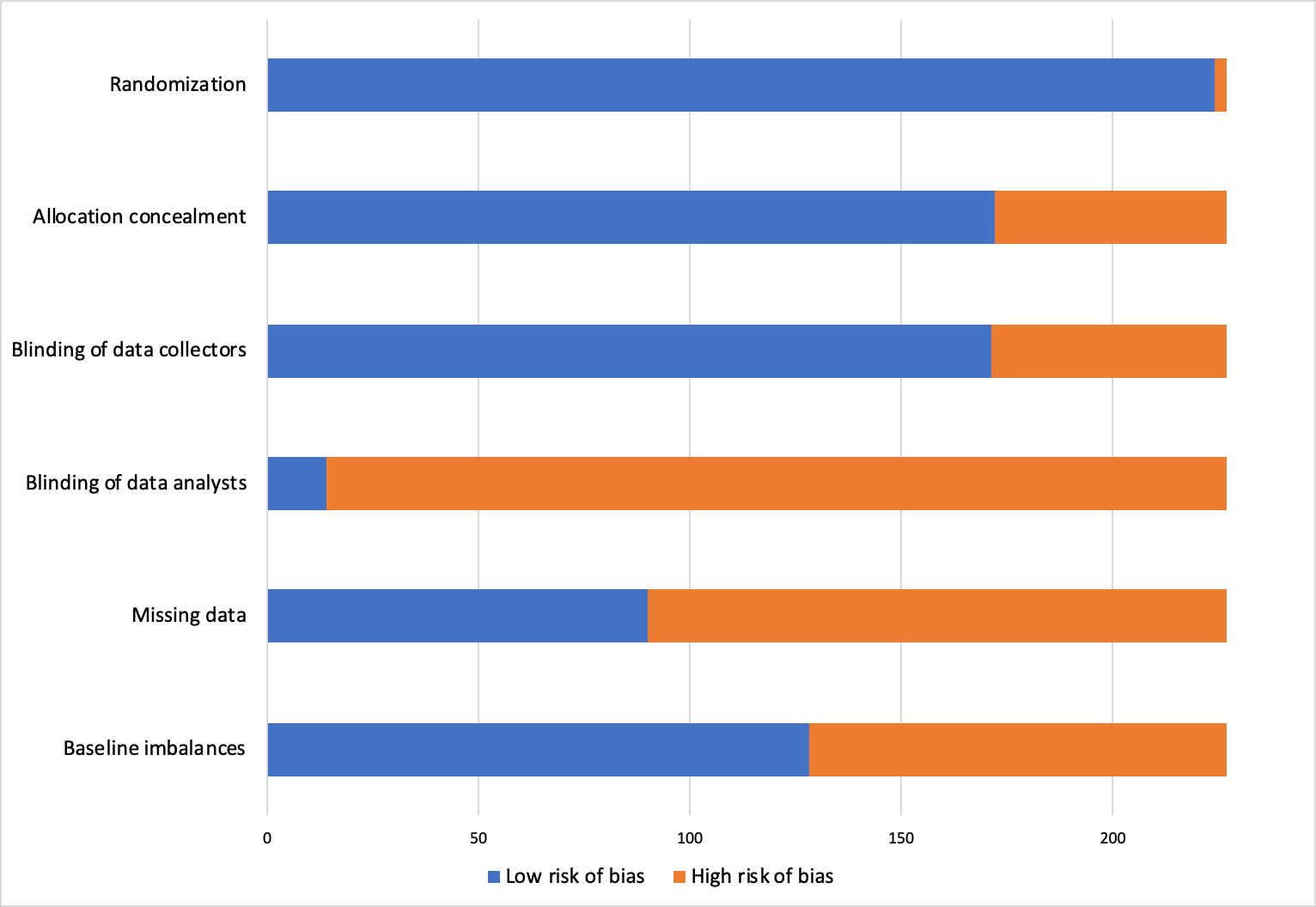


**eFigure 4.** Risk of bias inside intervention categories


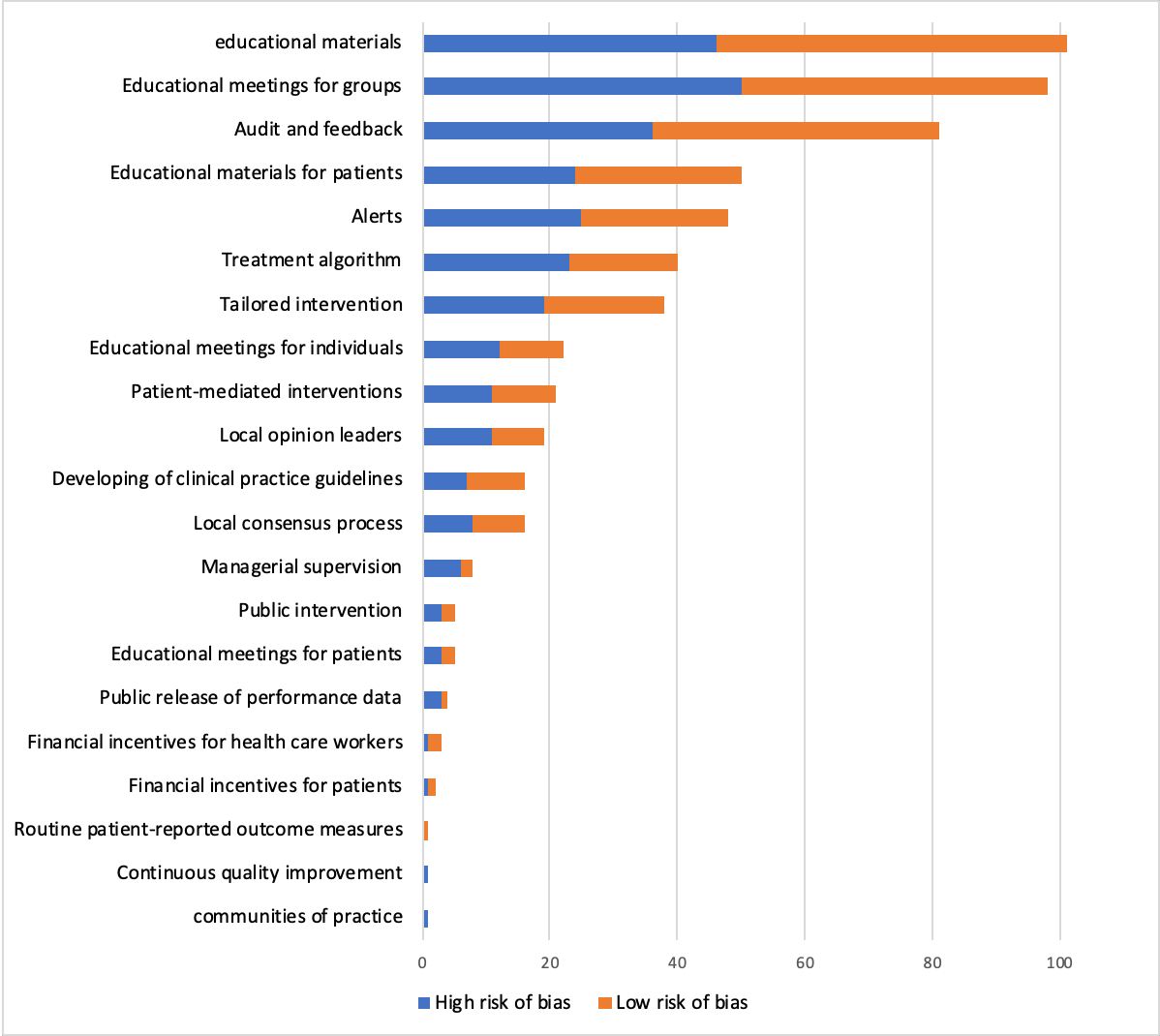


High risk of bias if three or under criteria judged as low risk of bias. Low risk of bias if four or more criteria assessed as low risk of bias.

**eFigure 5.** Intervention components in single-component interventions


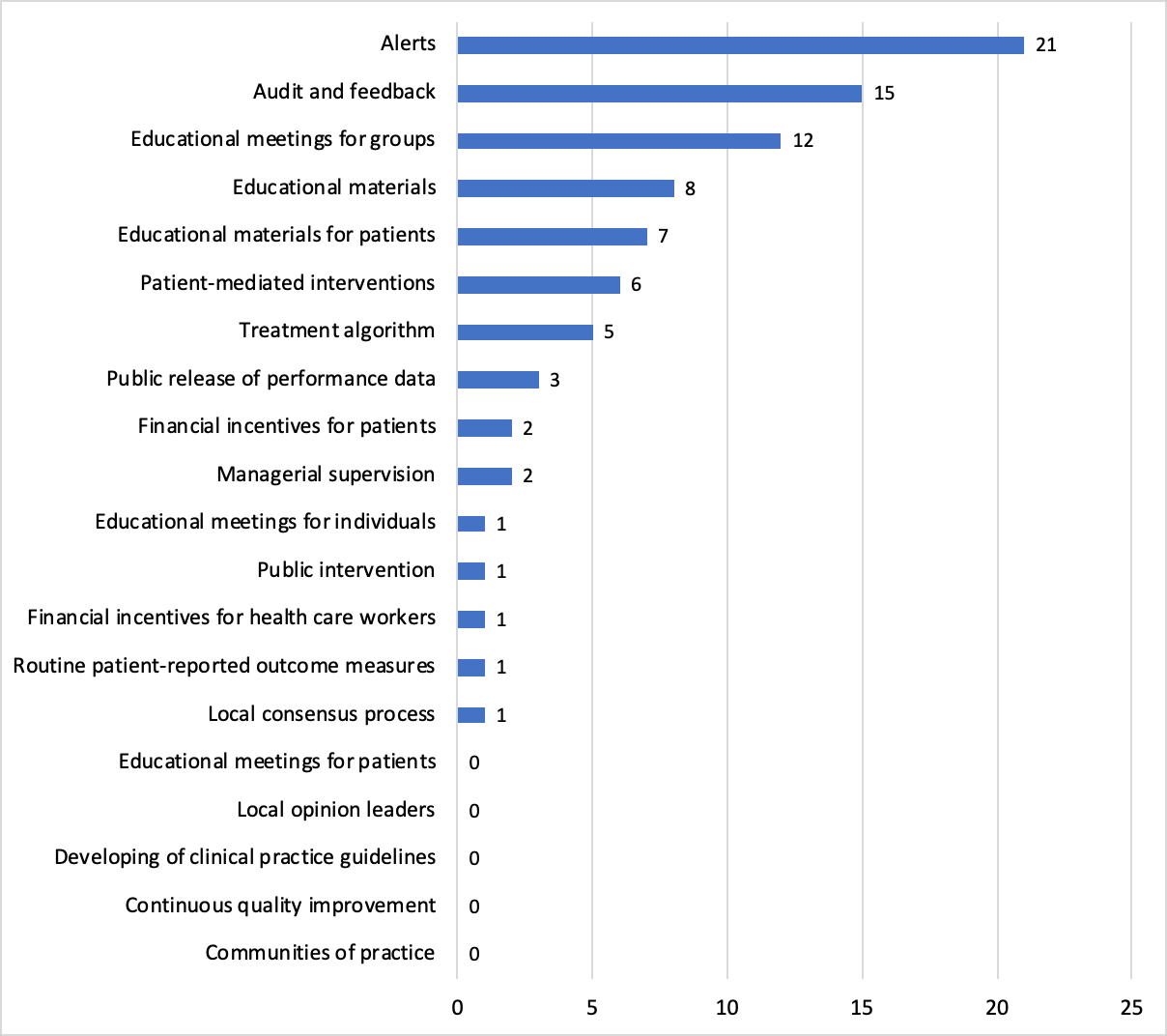


**eTable 1.** Quality outcomes until 2010 and after

|  | until 2010 | after 2010 |
| --- | --- | --- |
| Number of clusters (median) | 20 (IQR 31) | 30 (IQR 42) |
| Used ICC in sample size calculation | 19 (28%) | 31 (40%) |
| Follow up time (median) | 273 days | 335 days |
| Reported difference between baseline and after the intervention | 79 (71%) | 93 (81%) |
| Tailoring | 18 (16%) | 22 (19%) |
| Theoretical background | 21 (19%) | 27 (23%) |

## **eTable 2.** Theoretical background used in designing the interventions

## Classic theories

**Theory of planned behaviour**

Ajzen I. The theory of planned behaviour. Organ Behav Hum Decis Process 1991; 50: 179–211.

**Social cognitive theory**

Bandura A: Self-efficacy: the exercise of control. New York , Freeman; 1997

Bandura A. Social cognitive theory of self-regulation. Organ Behav Hum Decis Process 1991;50:248-8710. doi:1016/0749- 5978(91)90022-L

Bandura A. Social foundations of thought and action. Englewood Cliffs, NJ:PrenticeHall; 1986.

Maddux JE. Expectancies and the social cognitive perspective: basic principles, processes, and variables. In: Kirsch I, ed. How expectancies shape experiences. Washington, D.C.:American Psychological Association; 1999:17–40.

**Self determination theory**

Deci, E.L., & Ryan, R.M. (1980). The Empirical Exploration of Intrinsic Motivational Processes1. *Advances in Experimental Social Psychology, 13*, 39-80.

**stepwise care in behavioural change**

Kanfer FH, Goldstein AP (eds). Helping People Change. A textbook of methods. New York: Pergamon Press, 1986

**Nudge: Improving Decisions About Health, Wealth, and Happiness**

Thaler RH, Sunstein CR. Nudge: Improving Decisions About Health, Wealth, and Happiness. New York, NY: Penguin Books; 2009.

**Choises, values and frames**

Kahneman D, Tversky A. Choices, values and frames. Cambridge: Cambridge University Press; 2000.

**Heuristics and biases: the psychology of intuitive judgment**

Gilovich T, Griffin D, Kahneman D, editors. Heuristics and biases: the psychology of intuitive judgment. Cambridge: Cambridge University Press; 2002

**social learning theory**

Rotter JB. The Development and Applications of Social Learning Theory. New York, N.Y.: Praeger; 1982.

Rosenstock IM, Strecher VJ, Becker MH. Social learning theory and the Health Belief Model. Health Educ Q. 1988; 15: 175–183.

Rollnick S, Mason P, Butler CC. Health behaviour change: a guide for practitioners. Churchill Livingstone, 1999.

Butler CC, Rollnick S, Kinnersley P, Jones A, Stott NCH. Reducing antibiotics for
respiratory tract symptoms in primary care; consolidating “why” and considering “how.”
Br J Gen Pract 1998;48:1865-70.

**"Stages of Change" model**

Prochaska JO, Diclemente CC, Norcross JC: In search of how people change: applications to addictive behaviors. Am Psychol 1992; 47: 1102-1114

**Operant learning theory**

Eccles MP, Grimshaw JM, Johnston M et al. Applying psychological theories to evidence-based clinical practice: identifying factors predictive of managing upper respiratory tract infections
without antibiotics.Implement Sci 2007; 2: 26.

Blackman D: Operant conditioning: an experimental analysis of behaviour. Edited by: Blackman D. London , Methuen; 1974.

**Elaboration likelihood model of persuasion**

Petty RE, Cacioppo JT. The elaboration likelihood model of persuasion. In: Berkowitz L (ed). Advances in experimental social psychology. New York: Academic Press, 1986.

**Expectancy-valence theory**

Rotter JB. The Development and Applications of Social Learning Theory. New York, N.Y.: Praeger; 1982.

**Expectancy-value model**

Feather NT, ed. Expectations and action: expectancy-value models in psychology. Hillsdale, NJ: Lawrence Erlbaum Associates; 1982: 263–292

**Other cited theoretical literature**

Briñol P, Petty RE. Source factors in persuasion: a self-validation approach. Eur Rev Soc Psychol 2009; 20: 49–96.

Knights S. Reflection and learning: the importance of a listener. In: Boud D, Keogh R, Walker D, eds. Reflection: turning experience into learning. Kogan Page, 1985:85-90.

Yalom I. D. The Theory and Practice of Group Psychotherapy. Basic Books, New York, 1985

Bjork, RA. Retrieval practice and the maintenance of knowledge. In: Gruneberg, MM.; Morris, PE.; Sykes, RN., editors. Practical aspects of memory: current research and issues. New York: Wiley; 1988. p. 396-401.

Harden RM. Outcome-based education—The ostrich, the peacock and the beaver. Med Teach. 2007;29~7!:666– 671.

Halpern SD, Asch DA, Volpp KG. Commitment contracts as a way to health. BMJ 2012;344:e522.

Kazdin A. Behaviour modifi cation in applied settings, 6th edn. Belmont, CA: Wadsworth, 2001.

Mackie D. M., Worth L. T. and Asuncion A. G. Processing of persuasive in-group messages. J. Personality Sac. Psychol. 58, 812, 1990.

Weber A. L. Introduction to Psychology. Harper Collins, New York, 1991.

Karpicke JD, Roediger HL 3rd. The critical importance of retrieval for learning. Science 2008;319(5865):966–8. [PubMed: 18276894]

## Process models

Graham ID, Logan J, Harrison MB, et al. Lost in knowledge translation: time for a MAP? J Contin Educ Health Prof 2006;26:13–24.

## Evaluation frameworks

**Theoretical Domains Framework of behaviour change**

Cane J, O’Connor D, Michie S (2012) Validation of the theoretical domains framework for use in behaviour change and implementation research. Implement Sci 7: 37.

Michie S, Johnston M, Francis JJ, Hardeman W, Eccles M. From theory to intervention: mapping theoretically derived behavioural determinants to behaviour change techniques. Appl Psychol:An Inter Rev. 2008;57:660–80.

Michie S, Johnston M, Abraham C, Lawton R, Parker D, Walker A. Making psychological theory useful for implementing evidence based practice: a consensus approach. Qual Saf Health Care. 2005;14:26–33.

**Predisposing, Reinforcing and Enabling Constructs in Educational Diagnosis and Evaluation (PRECEDE) model of behavior change**

Green LW, Kreuter M. Health Program Planning: an Educational and Ecological Approach. New York: McGraw-Hill, 2005.

Mullen PD, Hersey JC, Iverson DC. Health behavior models compared. Soc Sci Med. 1987;24:973-981.

**Process evaluation on quality improvement interventions**

Hulscher ME, Laurant MG, Grol RP. Process evaluation on quality improvement interventions. Qual Saf Health Care 2003; 12: 40–6.

**Other**

Michie S, Richardson M, Johnston M, Abraham C, Francis J, Hardman W, et al. The behavior change technique taxonomy (v1) of 93 hierarchically clustered techniques: building an international consensus for the reporting of behavior change interventions. Ann Behav Med. 2013; 46:81–95. doi: 10.1007/ s12160-013-9486-6 PMID: 23512568

## Determinant frameworks

**Barriers and facilitators to implementing shared decision making in clinical practice: update of a systematic review of health professionals perceptions.**

Le´gare´ F, Ratte´ S, Gravel K, Graham ID. Barriers and facilitators to implementing shared decision making in clinical practice: update of a systematic review of health professionals perceptions. Patient Education and Counseling, 2008; 73: 526–535.

**A conceptual framework for understanding and reducing overuse by primary care providers**

Powell AA, Bloomfield HE, Burgess DJ, Wilt TJ, Partin MR. A Conceptual framework for understanding and reducing overuse by primary care providers. Med Care Res Rev. 2013;70(5):451–72.

## Implementation theory

**Knowledge-to-Action framework**

Graham ID, Logan J, Harrison MB, et al. Lost in knowledge translation: time for a map?. J Contin Educ Health Prof. 2006;26(1):13-24. doi:10.1002/chp.47

**Principles of educational outreach (“academic detailing”) to improve clinical decision making**

Soumerai SB, Avorn J. Principles of educational outreach (“academic detailing”) to improve clinical decision making. JAMA 1990;263:549–556

**Social learning theory and the health belief model**

Rosenstock IM, Strecher VJ, Becker MH. Social learning theory and the health belief model. Health Educ Q. 1988;15:175-183.

**Other theoretical articles cited**

Grol R. Implementing guidelines in general practice care. QHC 1992; 2: 184–91.

Lomas J. Teaching old (and not so old) docs new tricks: effective ways to implement research findings. In: Dunn EV, Norton PG, Stewart M, Tudiver F, Bass MJ, eds. Disseminating research/changing practice. London: Sage, 1994: 1–18.

Grol R, Baker R, Moss F. Quality improvement research: understanding the science of change in health care. Qual Saf Health Care 2002;11:110-1.

Yardley L, Morrison L, Bradbury K et al. The person-based approach to intervention development: application to digital health-related behavior change interventions. J Med Internet Res 2015; 17: e30.

Green LW, Kreuter MW. Health Program Planning: An Educational and Ecological Approach. 4th ed. New York, NY: McGraw-Hill; 2005

Rollnick S, Kinnersley P, Butler C. Context-bound communication skills training: development of a new method. Med Educ 2002;36:377-83

Elder JP, Ayala GX, Harris S. Theories and intervention approaches to health-behavior change in primary care. Am J Prev Med. 1999;17:275-284.

Harden RM. AMEE Guide No. 14: Outcome-based education: Part 1—An introduction to outcome-based education. Med Teach. 1999;21~1!:7–14.

Harden RM. Outcome-based education—The ostrich, the peacock and the beaver. Med Teach. 2007;29~7!:666– 671.

Raisch D. W. A model of methods for influencing prescribing: Part I--A review of prescribing models, persuasion theories, and administrative and educational methods. DICP 24, 417, 1990

Holmboe ES, Lipner R, Greiner A. Assessing quality of care: knowledge matters. JAMA 2008;299(3):338–40. [PubMed: 18212320]

IOM. Redesigning continuing education in the health professions. Washington DC: National
Academies Press; 2010.

Bennett NL, Davis DA, Easterling WE, Jr., Friedmann P, Green JS, Koeppen BM, Mazmanian PE, Waxman HS. Continuing medical education: A new vision of the professional development of physicians. Acad Med. 2000;75~12!:1167–1172.

Harrison R, Mitchell L. Using outcomes-based methodology for the education, training and assessment of competence of healthcare professionals. Med Teach. 2006;28~2!:165–170.

**eTable 3.** Citations for included studies

| **Agnew,​ J.,​Taaffe,​ M.,​Darker,​ C.,​O'Shea,​ B.,​Clarke,​ J.**. Delayed prescribing of antibiotics for respiratory tract infections: use of information leaflets. *Ir Med J.* 2013. 106:243-4 |
| --- |
| **Alder,​ Stephen C.,​Trunnell,​ Eric P.,​White,​ George L.,​Lyon,​ Joseph L.,​Reading,​ James P.,​Samore,​ Matthew H.,​Magill,​ Michael K.**. Reducing Parental Demand for Antibiotics by Promoting Communication Skills. *American Journal of Health Education.* 2005. 36:132-139 |
| **Alexander,​ E.,​Weingarten,​ S.,​Mohsenifar,​ Z.**. Clinical strategies to reduce utilization of chest physiotherapy without compromising patient care. *Chest.* 1996. 110:430-2 |
| **Alexandrino,​ A. S.,​Santos,​ R.,​Melo,​ C.,​Bastos,​ J. M.**. Impact of caregivers' education regarding respiratory infections on the health status of day-care children: a randomized trial. *Fam Pract.* 2016. 33:476-81 |
| **Althabe,​ F.,​Belizán,​ J. M.,​Villar,​ J.,​Alexander,​ S.,​Bergel,​ E.,​Ramos,​ S.,​Romero,​ M.,​Donner,​ A.,​Lindmark,​ G.,​Langer,​ A.,​Farnot,​ U.,​Cecatti,​ J. G.,​Carroli,​ G.,​Kestler,​ E.**. Mandatory second opinion to reduce rates of unnecessary caesarean sections in Latin America: A cluster randomised controlled trial. *Lancet.* 2004. 363:1934-1940 |
| **Altiner,​ Attila,​Brockmann,​ Silke,​Sielk,​ Martin,​Wilm,​ Stefan,​Wegscheider,​ Karl,​Abholz,​ Heinz-Harald**. Reducing antibiotic prescriptions for acute cough by motivating GPs to change their attitudes to communication and empowering patients: a cluster-randomized intervention study. *The Journal of antimicrobial chemotherapy.* 2007. 60:638-44 |
| **Anderson,​ J. F.,​McEwan,​ K. L.,​Hrudey,​ W. P.**. Effectiveness of notification and group education in modifying prescribing of regulated analgesics. *CMAJ : Canadian Medical Association journal = journal de l'Association medicale canadienne.* 1996. 154:31-9 |
| **Angunawela,​ I. I.,​Diwan,​ V. K.,​Tomson,​ G.**. Experimental evaluation of the effects of drug information on antibiotic prescribing: a study in outpatient care in an area of Sri lanka. *International journal of epidemiology.* 1991. 20:558-64 |
| **Ashworth,​ Nigel,​Kain,​ Nicole,​Wiebe,​ Delaney,​Hernandez-Ceron,​ Nancy,​Jess,​ Ed,​Mazurek,​ Karen**. Reducing prescribing of benzodiazepines in older adults: a comparison of four physician-focused interventions by a medical regulatory authority. *BMC family practice.* 2021. 22:68 |
| **Auleley,​ Guy-Robert,​Ravaud,​ Philippe,​Giraudeau,​ Bruno,​Kerboull,​ Luc,​Nizard,​ Rémy,​Massin,​ Philippe,​de Loubresse,​ Christian Garreau,​Vallée,​ Christian,​Durieux,​ Pierre**. Implementation of the Ottawa Ankle Rules in France: A Multicenter Randomized Controlled Trial. *JAMA.* 1997. 277:1935-1939 |
| **Avorn,​ J.,​Soumerai,​ S. B.**. Improving drug-therapy decisions through educational outreach. A randomized controlled trial of academically based "detailing". *The New England journal of medicine.* 1983. 308:1457-63 |
| **Awad,​ A. I.,​Eltayeb,​ I. B.,​Baraka,​ O. Z.**. Changing antibiotics prescribing practices in health centers of Khartoum State,​ Sudan. *European journal of clinical pharmacology.* 2006. 62:135-42 |
| **Bates,​ D. W.,​Kuperman,​ G. J.,​Jha,​ A.,​Teich,​ J. M.,​Orav,​ E. J.,​Ma'luf,​ N.,​Onderdonk,​ A.,​Pugatch,​ R.,​Wybenga,​ D.,​Winkelman,​ J.,​Brennan,​ T. A.,​Komaroff,​ A. L.,​Tanasijevic,​ M. J.**. Does the computerized display of charges affect inpatient ancillary test utilization?. *Archives of internal medicine.* 1997. 157:2501-8 |
| **Bates,​ D. W.,​Kuperman,​ G. J.,​Rittenberg,​ E.,​Teich,​ J. M.,​Fiskio,​ J.,​Ma'luf,​ N.,​Onderdonk,​ A.,​Wybenga,​ D.,​Winkelman,​ J.,​Brennan,​ T. A.,​Komaroff,​ A. L.,​Tanasijevic,​ M.**. A randomized trial of a computer-based intervention to reduce utilization of redundant laboratory tests. *The American journal of medicine.* 1999. 106:144-50 |
| **Berings,​ D.,​Blondeel,​ L.,​Habraken,​ H.**. The effect of industry-independent drug information on the prescribing of benzodiazepines in general practice. *Eur J Clin Pharmacol.* 1994. 46:501-5 |
| **Bernal-Delgado,​ E.,​Galeote-Mayor,​ M.,​Pradas-Arnal,​ F.,​Peiro-Moreno,​ S.**. Evidence based educational outreach visits: effects on prescriptions of non-steroidal anti-inflammatory drugs. *Journal of epidemiology and community health.* 2002. 56:653-8 |
| **Bexell,​ A.,​Lwando,​ E.,​von Hofsten,​ B.,​Tembo,​ S.,​Eriksson,​ B.,​Diwan,​ V. K.**. Improving drug use through continuing education: a randomized controlled trial in Zambia. *J Clin Epidemiol.* 1996. 49:355-7 |
| **Bhatia,​ R. S.,​Dudzinski,​ D. M.,​Malhotra,​ R.,​Milford,​ C. E.,​Yoerger Sanborn,​ D. M.,​Picard,​ M. H.,​Weiner,​ R. B.**. Educational intervention to reduce outpatient inappropriate echocardiograms: a randomized control trial. *JACC Cardiovasc Imaging.* 2014. 7:857-66 |
| **Bhatia,​ R. S.,​Ivers,​ N. M.,​Yin,​ X. C.,​Myers,​ D.,​Nesbitt,​ G. C.,​Edwards,​ J.,​Yared,​ K.,​Wadhera,​ R. K.,​Wu,​ J. C.,​Kithcart,​ A. P.,​Wong,​ B. M.,​Hansen,​ M. S.,​Weinerman,​ A. S.,​Shadowitz,​ S.,​Elman,​ D.,​Farkouh,​ M. E.,​Thavendiranathan,​ P.,​Udell,​ J. A.,​Johri,​ A. M.,​Chow,​ C. M.,​Hall,​ J.,​Bouck,​ Z.,​Cohen,​ A.,​Thorpe,​ K. E.,​Rakowski,​ H.,​Picard,​ M. H.,​Weiner,​ R. B.**. Improving the Appropriate Use of Transthoracic Echocardiography: The Echo WISELY Trial. *Journal of the American College of Cardiology.* 2017. 70:1135-1144 |
| **Bhunia,​ R.,​Hutin,​ Y.,​Ramkrishnan,​ R.,​Ghosh,​ P. K.,​Dey,​ S.,​Murhekar,​ M.**. Reducing use of injections through interactional group discussions: a randomized controlled trial. *Indian pediatrics.* 2010. 47:409-14 |
| **Bindels,​ R.,​Hasman,​ A.,​Kester,​ A.,​Talmon,​ J. L.,​de Clercq,​ P. A.,​Winkens,​ R. A. G.**. The efficacy of an automated feedback system for general practitioners. *Informatics in Primary Care.* 2003. 11:69-74 |
| **Blair,​ Peter S.,​Turnbull,​ Sophie,​Ingram,​ Jenny,​Redmond,​ Niamh,​Lucas,​ Patricia Jane,​Cabral,​ Christie,​Hollinghurst,​ Sandra,​Dixon,​ Padraig,​Peters,​ Tim,​Horwood,​ Jeremy,​Little,​ Paul,​Francis,​ Nick A.,​Gilbertson,​ Anna,​Jameson,​ Catherine,​Hay,​ Alastair D.**. Feasibility cluster randomised controlled trial of a within-consultation intervention to reduce antibiotic prescribing for children presenting to primary care with acute respiratory tract infection and cough. *BMJ open.* 2017. 7:e014506 |
| **Boulet,​ L.,​Vermeulin,​ T.,​Vasiliu,​ A.,​Gillibert,​ A.,​Lottin,​ M.,​Frébourg,​ N.,​Boyer,​ S.,​Merle,​ V.**. Lack of effect of a poster-based intervention to reduce the number of blood culture samples collected. *Medecine et Maladies Infectieuses.* 2019. #volume#:#pages# |
| **Bourgeois,​ Fabienne C.,​Linder,​ Jeffrey,​Johnson,​ Sarah A.,​Co,​ John Patrick T.,​Fiskio,​ Julie,​Ferris,​ Timothy G.**. Impact of a computerized template on antibiotic prescribing for acute respiratory infections in children and adolescents. *Clinical pediatrics.* 2010. 49:976-83 |
| **Briel,​ Matthias,​Langewitz,​ Wolf,​Tschudi,​ Peter,​Young,​ Jim,​Hugenschmidt,​ Christa,​Bucher,​ Heiner C.**. Communication training and antibiotic use in acute respiratory tract infections. A cluster randomised controlled trial in general practice. *Swiss medical weekly.* 2006. 136:241-7 |
| **Bunt,​ Christopher W.,​Burke,​ Harry B.,​Towbin,​ Alexander J.,​Hoang,​ Albert,​Stephens,​ Mark B.,​Fontelo,​ Paul,​Liu,​ Fang,​Gimbel,​ Ronald W.**. Point-of-Care Estimated Radiation Exposure and Imaging Guidelines Can Reduce Pediatric Radiation Burden. *Journal of the American Board of Family Medicine : JABFM.* 2015. 28:343-50 |
| **Butler,​ Christopher C.,​Simpson,​ Sharon A.,​Dunstan,​ Frank,​Rollnick,​ Stephen,​Cohen,​ David,​Gillespie,​ David,​Evans,​ Meirion R.,​Alam,​ M. Fasihul,​Bekkers,​ Marie-Jet,​Evans,​ John,​Moore,​ Laurence,​Howe,​ Robin,​Hayes,​ Jamie,​Hare,​ Monika,​Hood,​ Kerenza**. Effectiveness of multifaceted educational programme to reduce antibiotic dispensing in primary care: practice based randomised controlled trial. *BMJ (Clinical research ed.).* 2012. 344:d8173 |
| **Cals,​ Jochen W. L.,​Butler,​ Christopher C.,​Hopstaken,​ Rogier M.,​Hood,​ Kerenza,​Dinant,​ Geert-Jan**. Effect of point of care testing for C reactive protein and training in communication skills on antibiotic use in lower respiratory tract infections: cluster randomised trial. *BMJ (Clinical research ed.).* 2009. 338:b1374  **Cals,​ Jochen W. L.,​Ament,​ Andre J. H. A.,​Hood,​ Kerenza,​Butler,​ Christopher C.,​Hopstaken,​ Rogier M.,​Wassink,​ Geert F.,​Dinant,​ Geert-Jan**. C-reactive protein point of care testing and physician communication skills training for lower respiratory tract infections in general practice: economic evaluation of a cluster randomized trial. *Journal of evaluation in clinical practice.* 2011. 17:1059-69  **Cals,​ Jochen W. L.,​de Bock,​ Leon,​Beckers,​ Pieter-Jan H. W.,​Francis,​ Nick A.,​Hopstaken,​ Rogier M.,​Hood,​ Kerenza,​de Bont,​ Eefje G. P. M.,​Butler,​ Christopher C.,​Dinant,​ Geert-Jan**. Enhanced communication skills and C-reactive protein point-of-care testing for respiratory tract infection: 3.5-year follow-up of a cluster randomized trial. *Annals of family medicine.* 2013. 11:157-64 |
| **Chaillet,​ N.,​Dumont,​ A.,​Abrahamowicz,​ M.,​Pasquier,​ J. C.,​Audibert,​ F.,​Monnier,​ P.,​Abenhaim,​ H. A.,​Dubé,​ E.,​Dugas,​ M.,​Burne,​ R.,​Fraser,​ W. D.**. A cluster-randomized trial to reduce cesarean delivery rates in Quebec. *New England Journal of Medicine.* 2015. 372:1710-1721 |
| **Champion,​ Karine,​Mouly,​ Stephane,​Lloret-Linares,​ Celia,​Lopes,​ Amanda,​Vicaut,​ Eric,​Bergmann,​ Jean-Francois,​Permi Investigators Committee**. Optimizing the use of intravenous therapy in internal medicine. *The American journal of medicine.* 2013. 126:925.e1-9 |
| **Chang,​ Yue,​ Sangthong,​ Rassamee,​ McNeil,​ Edward B.,​ Tang,​ Lei,​ Chongsuvivatwong,​ Virasakdi**. Effect of a computer network-based feedback program on antibiotic prescription rates of primary care physicians: A cluster randomized crossover-controlled trial. *Journal of infection and public health.* 2020. #volume#:#pages# |
| **Chassin,​ M. R.,​McCue,​ S. M.**. A randomized trial of medical quality assurance. Improving physicians' use of pelvimetry. *JAMA.* 1986. 256:1012-6 |
| **Chazan,​ Bibiana,​Turjeman,​ Ruth Ben Zur,​Frost,​ Yosef,​Besharat,​ Beshara,​Tabenkin,​ Hava,​Stainberg,​ Avi,​Sakran,​ Waheeb,​Raz,​ Raul**. Antibiotic consumption successfully reduced by a community intervention program. *The Israel Medical Association journal : IMAJ.* 2007. 9:16-20 |
| **Chien,​ Alyna T.,​Lehmann,​ Lisa Soleymani,​Hatfield,​ Laura A.,​Koplan,​ Kate E.,​Petty,​ Carter R.,​Sinaiko,​ Anna D.,​Rosenthal,​ Meredith B.,​Sequist,​ Thomas D.**. A Randomized Trial of Displaying Paid Price Information on Imaging Study and Procedure Ordering Rates. *Journal of general internal medicine.* 2017. 32:434-448 |
| **Chin,​ K. K.,​ Svec,​ D.,​ Leung,​ B.,​ Sharp,​ C.,​ Shieh,​ L.**. E-HeaRT BPA: Electronic health record telemetry BPA. *Postgraduate Medical Journal.* 2020. #volume#:#pages# |
| **Christakis,​ D. A.,​Zimmerman,​ F. J.,​Wright,​ J. A.,​Garrison,​ M. M.,​Rivara,​ F. P.,​Davis,​ R. L.**. A randomized controlled trial of point-of-care evidence to improve the antibiotic prescribing practices for otitis media in children. *Pediatrics.* 2001. 107:E15 |
| **Coenen,​ S.,​Van Royen,​ P.,​Michiels,​ B.,​Denekens,​ J.**. Optimizing antibiotic prescribing for acute cough in general practice: a cluster-randomized controlled trial. *J Antimicrob Chemother.* 2004. 54:661-72 |
| **Cohen,​ R.,​Allaert,​ F. A.,​Callens,​ A.,​Menn,​ S.,​Urbinelli,​ R.,​Roden,​ A.**. Medico-economic evaluation of an educational intervention to optimize children uncomplicated nasopharyngitis treatment in ambulatory care. *Medecine et Maladies Infectieuses.* 2000. 30:691-698 |
| **Coombs,​ Danielle M.,​Machado,​ Gustavo C.,​Richards,​ Bethan,​Needs,​ Chris,​Buchbinder,​ Rachelle,​Harris,​ Ian A.,​Howard,​ Kirsten,​McCaffery,​ Kirsten,​Billot,​ Laurent,​Edwards,​ James,​Rogan,​ Eileen,​Facer,​ Rochelle,​Li,​ Qiang,​Maher,​ Christopher G.**. Effectiveness of a multifaceted intervention to improve emergency department care of low back pain: a stepped-wedge,​ cluster-randomised trial. *BMJ quality & safety.* 2021. #volume#:#pages# |
| **Cummings,​ K. M.,​Frisof,​ K. B.,​Long,​ M. J.,​Hrynkiewich,​ G.**. The effects of price information on physicians' test-ordering behavior. Ordering of diagnostic tests. *Med Care.* 1982. 20:293-301 |
| **Cundill,​ Bonnie,​Mbakilwa,​ Hilda,​Chandler,​ Clare Ir,​Mtove,​ George,​Mtei,​ Frank,​Willetts,​ Annie,​Foster,​ Emily,​Muro,​ Florida,​Mwinyishehe,​ Rahim,​Mandike,​ Renata,​Olomi,​ Raimos,​Whitty,​ Christopher Jm,​Reyburn,​ Hugh**. Prescriber and patient-oriented behavioural interventions to improve use of malaria rapid diagnostic tests in Tanzania: facility-based cluster randomised trial. *BMC medicine.* 2015. 13:118 |
| **Curtis,​ Helen J.,​Bacon,​ Seb,​Croker,​ Richard,​Walker,​ Alex J.,​Perera,​ Rafael,​Hallsworth,​ Michael,​Harper,​ Hugo,​Mahtani,​ Kamal R.,​Heneghan,​ Carl,​Goldacre,​ Ben**. Evaluating the impact of a very low-cost intervention to increase practices' engagement with data and change prescribing behaviour: a randomized trial in English primary care. *Family practice.* 2021. #volume#:#pages# |
| **Daley,​ Peter,​Garcia,​ David,​Inayatullah,​ Raheel,​Penney,​ Carla,​Boyd,​ Sarah**. Modified Reporting of Positive Urine Cultures to Reduce Inappropriate Treatment of Asymptomatic Bacteriuria Among Nonpregnant,​ Noncatheterized Inpatients: A Randomized Controlled Trial. *Infection control and hospital epidemiology.* 2018. 39:814-819 |
| **Danaher,​ P. J.,​Milazzo,​ N. A.,​Kerr,​ K. J.,​Lagasse,​ C. A.,​Lane,​ J. W.**. The antibiotic support team--a successful educational approach to antibiotic stewardship. *Mil Med.* 2009. 174:201-5 |
| **Daneman,​ Nick,​Lee,​ Samantha M.,​Bai,​ Heming,​Bell,​ Chaim M.,​Bronskill,​ Susan E.,​Campitelli,​ Michael A.,​Dobell,​ Gail,​Fu,​ Longdi,​Garber,​ Gary,​Ivers,​ Noah,​Lam,​ Jonathan M. C.,​Langford,​ Bradley J.,​Laur,​ Celia,​Morris,​ Andrew,​Mulhall,​ Cara,​Pinto,​ Ruxandra,​Saxena,​ Farah E.,​Schwartz,​ Kevin L.,​Brown,​ Kevin A.**. Population-Wide Peer Comparison Audit and Feedback to Reduce Antibiotic Initiation and Duration in Long-Term Care Facilities with Embedded Randomized Controlled Trial. *Clinical infectious diseases : an official publication of the Infectious Diseases Society of America.* 2021. #volume#:#pages# |
| **Das,​ Jishnu,​Chowdhury,​ Abhijit,​Hussam,​ Reshmaan,​Banerjee,​ Abhijit V.**. The impact of training informal health care providers in India: A randomized controlled trial. *Science (New York,​ N.Y.).* 2016. 354:#pages# |
| **Davidoff, F.,Goodspeed, R.,Clive, J.**. Changing test ordering behavior. A randomized controlled trial comparing probabilistic reasoning with cost-containment education. *Medical care.* 1989. 27:45-58 |
| **de Bont,​ Eefje G. P. M.,​Dinant,​ Geert-Jan,​Elshout,​ Gijs,​van Well,​ Gijs,​Francis,​ Nick A.,​Winkens,​ Bjorn,​Cals,​ Jochen W. L.**. Booklet for Childhood Fever in Out-of-Hours Primary Care: A Cluster-Randomized Controlled Trial. *Annals of family medicine.* 2018. 16:314-321 |
| **de Burgh,​ S.,​Mant,​ A.,​Mattick,​ R. P.,​Donnelly,​ N.,​Hall,​ W.,​Bridges-Webb,​ C.**. A controlled trial of educational visiting to improve benzodiazepine prescribing in general practice. *Australian journal of public health.* 1995. 19:142-8  **Mant,​ A.,​de Burgh,​ S.,​Mattick,​ R. P.,​Donnelly,​ N.,​Hall,​ W.**. Insomnia in general practice. Results from NSW General Practice Survey 1991-1992. *Australian family physician.* 1996. Suppl 1:S15-18 |
| **Dekker,​ Anne R. J.,​Verheij,​ Theo J. M.,​Broekhuizen,​ Berna D. L.,​Butler,​ Christopher C.,​Cals,​ Jochen W. L.,​Francis,​ Nick A.,​Little,​ Paul,​Sanders,​ Elisabeth A. M.,​Yardley,​ Lucy,​Zuithoff,​ Nicolaas P. A.,​van der Velden,​ Alike W.**. Effectiveness of general practitioner online training and an information booklet for parents on antibiotic prescribing for children with respiratory tract infection in primary care: a cluster randomized controlled trial. *The Journal of antimicrobial chemotherapy.* 2018. 73:1416-1422 |
| **Dey,​ Paola,​Simpson,​ Carl W. R.,​Collins,​ Stuart I.,​Hodgson,​ G.,​Dowrick,​ Christopher F.,​Simison,​ A. J. M.,​Rose,​ M. J.**. Implementation of RCGP guidelines for acute low back pain: a cluster randomised controlled trial. *The British journal of general practice : the journal of the Royal College of General Practitioners.* 2004. 54:33-37 |
| **Dormuth,​ Colin R.,​Carney,​ Greg,​Taylor,​ Suzanne,​Bassett,​ Ken,​Maclure,​ Malcolm**. A randomized trial assessing the impact of a personal printed feedback portrait on statin prescribing in primary care. *The Journal of continuing education in the health professions.* 2012. 32:153-62 |
| **Doyne,​ Emanuel O.,​Alfaro,​ Mary Pat,​Siegel,​ Robert M.,​Atherton,​ Harry D.,​Schoettker,​ Pamela J.,​Bernier,​ Jeralyn,​Kotagal,​ Uma R.**. A randomized controlled trial to change antibiotic prescribing patterns in a community. *Archives of pediatrics & adolescent medicine.* 2004. 158:577-83 |
| **Dreischulte,​ Tobias,​Donnan,​ Peter,​Grant,​ Aileen,​Hapca,​ Adrian,​McCowan,​ Colin,​Guthrie,​ Bruce**. Safer Prescribing--A Trial of Education,​ Informatics,​ and Financial Incentives. *The New England journal of medicine.* 2016. 374:1053-64 |
| **Dudzinski,​ David M.,​Bhatia,​ R. Sacha,​Mi,​ Michael Y.,​Isselbacher,​ Eric M.,​Picard,​ Michael H.,​Weiner,​ Rory B.**. Effect of Educational Intervention on the Rate of Rarely Appropriate Outpatient Echocardiograms Ordered by Attending Academic Cardiologists: A Randomized Clinical Trial. *JAMA cardiology.* 2016. 1:805-812 |
| **Du Yan, Lily,Dean, Kristin,Park, Daniel,Thompson, James,Tong, Ian,Liu, Cindy,Hamdy, Rana F.**. Education vs Clinician Feedback on Antibiotic Prescriptions for Acute Respiratory Infections in Telemedicine: a Randomized Controlled Trial. *Journal of general internal medicine.* 2021. 36:305-312 |
| **Eccles,​ M.,​Steen,​ N.,​Grimshaw,​ J.,​Thomas,​ L.,​McNamee,​ P.,​Soutter,​ J.,​Wilsdon,​ J.,​Matowe,​ L.,​Needham,​ G.,​Gilbert,​ F.,​Bond,​ S.**. Effect of audit and feedback,​ and reminder messages on primary-care radiology referrals: a randomised trial. *Lancet (London,​ England).* 2001. 357:1406-9 |
| **Egan,​ Katie G.,​ De Souza,​ Michelle,​ Muenks,​ Elizabeth,​ Nazir,​ Niaman,​ Korentager,​ Richard**. Opioid Consumption Following Breast Surgery Decreases with a Brief Educational Intervention: A Randomized,​ Controlled Trial. *Annals of surgical oncology.* 2020. 27:3156-3162 |
| **Eilermann,​ Kerstin,​Halstenberg,​ Katrin,​Kuntz,​ Ludwig,​Martakis,​ Kyriakos,​Roth,​ Bernhard,​Wiesen,​ Daniel**. The Effect of Expert Feedback on Antibiotic Prescribing in Pediatrics: Experimental Evidence. *Medical decision making : an international journal of the Society for Medical Decision Making.* 2019. 39:781-795 |
| **Elouafkaoui,​ Paula,​Young,​ Linda,​Newlands,​ Rumana,​Duncan,​ Eilidh M.,​Elders,​ Andrew,​Clarkson,​ Jan E.,​Ramsay,​ Craig R.,​Translation Research in a Dental Setting Research Methodology,​ Group**. An Audit and Feedback Intervention for Reducing Antibiotic Prescribing in General Dental Practice: The RAPiD Cluster Randomised Controlled Trial. *PLoS medicine.* 2016. 13:e1002115 |
| **Eltayeb,​ I. B.,​Awad,​ A. I.,​Mohamed-Salih,​ M. S.,​Daffa-Alla,​ M. A.,​Ahmed,​ M. B.,​Ogail,​ M. A.,​Matowe,​ L.**. Changing the prescribing patterns of sexually transmitted infections in the White Nile Region of Sudan. *Sexually transmitted infections.* 2005. 81:426-7 |
| **English,​ D. R.,​Burton,​ R. C.,​Del Mar,​ C. B.,​Donovan,​ R. J.,​Ireland,​ P. D.,​Emery,​ G.**. Evaluation of aid to diagnosis of pigmented skin lesions in general practice: Controlled trial randomised by practice. *British Medical Journal.* 2003. 327:375-378 |
| **Esmaily,​ H. M.,​Silver,​ I.,​Shiva,​ S.,​Gargani,​ A.,​Maleki-Dizaji,​ N.,​Al-Maniri,​ A.,​Wahlstrom,​ R.**. Can rational prescribing be improved by an outcome-based educational approach? A randomized trial completed in Iran. *Journal of Continuing Education in the Health Professions.* 2010. 30:11-18 |
| **Evans,​ L. K.,​Strumpf,​ N. E.,​Allen-Taylor,​ S. L.,​Capezuti,​ E.,​Maislin,​ G.,​Jacobsen,​ B.**. A clinical trial to reduce restraints in nursing homes. *J Am Geriatr Soc.* 1997. 45:675-81 |
| **Feldman,​ Leonard S.,​Shihab,​ Hasan M.,​Thiemann,​ David,​Yeh,​ Hsin-Chieh,​Ardolino,​ Margaret,​Mandell,​ Steven,​Brotman,​ Daniel J.**. Impact of providing fee data on laboratory test ordering: a controlled clinical trial. *JAMA internal medicine.* 2013. 173:903-8 |
| **Fenton,​ Joshua J.,​Kravitz,​ Richard L.,​Jerant,​ Anthony,​Paterniti,​ Debora A.,​Bang,​ Heejung,​Williams,​ Donna,​Epstein,​ Ronald M.,​Franks,​ Peter**. Promoting Patient-Centered Counseling to Reduce Use of Low-Value Diagnostic Tests: A Randomized Clinical Trial. *JAMA internal medicine.* 2016. 176:191-7 |
| **Fine,​ M. J.,​Stone,​ R. A.,​Lave,​ J. R.,​Hough,​ L. J.,​Obrosky,​ D. S.,​Mor,​ M. K.,​Kapoor,​ W. N.**. Implementation of an evidence-based guideline to reduce duration of intravenous antibiotic therapy and length of stay for patients hospitalized with community-acquired pneumonia: a randomized controlled trial. *Am J Med.* 2003. 115:343-51 |
| **Finkelstein,​ J. A.,​Davis,​ R. L.,​Dowell,​ S. F.,​Metlay,​ J. P.,​Soumerai,​ S. B.,​Rifas-Shiman,​ S. L.,​Higham,​ M.,​Miller,​ Z.,​Miroshnik,​ I.,​Pedan,​ A.,​Platt,​ R.**. Reducing antibiotic use in children: a randomized trial in 12 practices. *Pediatrics.* 2001. 108:1-7 |
| **Finkelstein,​ Jonathan A.,​Huang,​ Susan S.,​Kleinman,​ Ken,​Rifas-Shiman,​ Sheryl L.,​Stille,​ Christopher J.,​Daniel,​ James,​Schiff,​ Nancy,​Steingard,​ Ron,​Soumerai,​ Stephen B.,​Ross-Degnan,​ Dennis,​Goldmann,​ Donald,​Platt,​ Richard**. Impact of a 16-community trial to promote judicious antibiotic use in Massachusetts. *Pediatrics.* 2008. 121:e15-23 |
| **Fleet,​ Elizabeth,​Gopal Rao,​ G.,​Patel,​ Bharat,​Cookson,​ Barry,​Charlett,​ Andre,​Bowman,​ Clive,​Davey,​ Peter**. Impact of implementation of a novel antimicrobial stewardship tool on antibiotic use in nursing homes: a prospective cluster randomized control pilot study. *The Journal of antimicrobial chemotherapy.* 2014. 69:2265-73 |
| **Flottorp,​ S.,​Oxman,​ A. D.,​Håvelsrud,​ K.,​Treweek,​ S.,​Herrin,​ J.**. Cluster randomised controlled trial of tailored interventions to improve the management of urinary tract infections in women and sore throat. *Bmj.* 2002. 325:367 |
| **Fortuna,​ Robert J.,​Zhang,​ Fang,​Ross-Degnan,​ Dennis,​Campion,​ Francis X.,​Finkelstein,​ Jonathan A.,​Kotch,​ Jamie B.,​Feldstein,​ Adrianne C.,​Smith,​ David H.,​Simon,​ Steven R.**. Reducing the prescribing of heavily marketed medications: a randomized controlled trial. *Journal of general internal medicine.* 2009. 24:897-903 |
| **Foxman B, Valdez RB, Lohr KN, Goldberg GA, Newhouse JP, Brook RH.** The effect of cost sharing on the use of antibiotics in ambulatory care: results from a population-based randomized controlled trial. *J Chronic Dis*. 1987;40(5):429-437. doi:10.1016/0021-9681(87)90176-7 |
| **Francis,​ Nick A.,​Butler,​ Christopher C.,​Hood,​ Kerenza,​Simpson,​ Sharon,​Wood,​ Fiona,​Nuttall,​ Jacqueline**. Effect of using an interactive booklet about childhood respiratory tract infections in primary care consultations on reconsulting and antibiotic prescribing: a cluster randomised controlled trial. *BMJ (Clinical research ed.).* 2009. 339:b2885 |
| **French,​ Simon D.,​McKenzie,​ Joanne E.,​O'Connor,​ Denise A.,​Grimshaw,​ Jeremy M.,​Mortimer,​ Duncan,​Francis,​ Jill J.,​Michie,​ Susan,​Spike,​ Neil,​Schattner,​ Peter,​Kent,​ Peter,​Buchbinder,​ Rachelle,​Page,​ Matthew J.,​Green,​ Sally E.**. Evaluation of a theory-informed implementation intervention for the management of acute low back pain in general medical practice: the IMPLEMENT cluster randomised trial. *PloS one.* 2013. 8:e65471 |
| **Gerber,​ Jeffrey S.,​Prasad,​ Priya A.,​Fiks,​ Alexander G.,​Localio,​ A. Russell,​Grundmeier,​ Robert W.,​Bell,​ Louis M.,​Wasserman,​ Richard C.,​Keren,​ Ron,​Zaoutis,​ Theoklis E.**. Effect of an outpatient antimicrobial stewardship intervention on broad-spectrum antibiotic prescribing by primary care pediatricians: a randomized trial. *JAMA.* 2013. 309:2345-52  **Gerber,​ J. S.,​Prasad,​ P. A.,​Fiks,​ A. G.,​Localio,​ A. R.,​Bell,​ L. M.,​Keren,​ R.,​Zaoutis,​ T. E.**. Durability of benefits of an outpatient antimicrobial stewardship intervention after discontinuation of audit and feedback. *JAMA - Journal of the American Medical Association.* 2014. 312:2569-2570 |
| **Gjelstad,​ Svein,​Hoye,​ Sigurd,​Straand,​ Jorund,​Brekke,​ Mette,​Dalen,​ Ingvild,​Lindbaek,​ Morten**. Improving antibiotic prescribing in acute respiratory tract infections: cluster randomised trial from Norwegian general practice (prescription peer academic detailing (Rx-PAD) study). *BMJ (Clinical research ed.).* 2013. 347:f4403 |
| **Goldberg,​ H. I.,​Deyo,​ R. A.,​Taylor,​ V. M.,​Cheadle,​ A. D.,​Conrad,​ D. A.,​Loeser,​ J. D.,​Heagerty,​ P. J.,​Diehr,​ P.**. Can evidence change the rate of back surgery? A randomized trial of community-based education. *Effective clinical practice : ECP.* 2001. 4:95-104 |
| **Gonzales,​ Ralph,​Anderer,​ Tammy,​McCulloch,​ Charles E.,​Maselli,​ Judith H.,​Bloom,​ Frederick J.,​ Jr.,​Graf,​ Thomas R.,​Stahl,​ Melissa,​Yefko,​ Michelle,​Molecavage,​ Julie,​Metlay,​ Joshua P.**. A cluster randomized trial of decision support strategies for reducing antibiotic use in acute bronchitis. *JAMA internal medicine.* 2013. 173:267-73 |
| **Gulliford,​ Martin C.,​Prevost,​ A. Toby,​Charlton,​ Judith,​Juszczyk,​ Dorota,​Soames,​ Jamie,​McDermott,​ Lisa,​Sultana,​ Kirin,​Wright,​ Mark,​Fox,​ Robin,​Hay,​ Alastair D.,​Little,​ Paul,​Moore,​ Michael V.,​Yardley,​ Lucy,​Ashworth,​ Mark**. Effectiveness and safety of electronically delivered prescribing feedback and decision support on antibiotic use for respiratory illness in primary care: REDUCE cluster randomised trial. *BMJ (Clinical research ed.).* 2019. 364:l236 |
| **Gulliford,​ Martin C.,​van Staa,​ Tjeerd,​Dregan,​ Alex,​McDermott,​ Lisa,​McCann,​ Gerard,​Ashworth,​ Mark,​Charlton,​ Judith,​Little,​ Paul,​Moore,​ Michael V.,​Yardley,​ Lucy**. Electronic health records for intervention research: a cluster randomized trial to reduce antibiotic prescribing in primary care (eCRT study). *Annals of family medicine.* 2014. 12:344-51 |
| **Hadiyono,​ J. E.,​Suryawati,​ S.,​Danu,​ S. S.,​Sunartono,​,​Santoso,​ B.**. Interactional group discussion: results of a controlled trial using a behavioral intervention to reduce the use of injections in public health facilities. *Social science & medicine (1982).* 1996. 42:1177-83 |
| **Hallsworth,​ Michael,​Chadborn,​ Tim,​Sallis,​ Anna,​Sanders,​ Michael,​Berry,​ Daniel,​Greaves,​ Felix,​Clements,​ Lara,​Davies,​ Sally C.**. Provision of social norm feedback to high prescribers of antibiotics in general practice: a pragmatic national randomised controlled trial. *Lancet (London,​ England).* 2016. 387:1743-52 |
| **Hamilton,​ William,​Russell,​ David,​Stabb,​ Catherine,​Seamark,​ David,​Campion-Smith,​ Charles,​Britten,​ Nicky**. The effect of patient self-completion agenda forms on prescribing and adherence in general practice: a randomized controlled trial. *Family practice.* 2007. 24:77-83 |
| **Haskell,​ Libby,​Tavender,​ Emma J.,​Wilson,​ Catherine L.,​O'Brien,​ Sharon,​Babl,​ Franz E.,​Borland,​ Meredith L.,​Cotterell,​ Elizabeth,​Schembri,​ Rachel,​Orsini,​ Francesca,​Sheridan,​ Nicolette,​Johnson,​ David W.,​Oakley,​ Ed,​Dalziel,​ Stuart R.,​Predict Network**. Effectiveness of Targeted Interventions on Treatment of Infants With Bronchiolitis: A Randomized Clinical Trial. *JAMA pediatrics.* 2021. #volume#:#pages# |
| **Hemkens,​ Lars G.,​Saccilotto,​ Ramon,​Reyes,​ Selene Leon,​Glinz,​ Dominik,​Zumbrunn,​ Thomas,​Grolimund,​ Oliver,​Gloy,​ Viktoria,​Raatz,​ Heike,​Widmer,​ Andreas,​Zeller,​ Andreas,​Bucher,​ Heiner C.**. Personalized Prescription Feedback Using Routinely Collected Data to Reduce Antibiotic Use in Primary Care: A Randomized Clinical Trial. *JAMA internal medicine.* 2017. 177:176-183 |
| **Hemminki,​ Elina,​Heikkilä,​ Kaija,​Sevón,​ Tiina,​Koponen,​ Päivikki**. Special features of health services and register based trials – experiences from a randomized trial of childbirth classes. *BMC Health Services Research.* 2008. 8:126 |
| **Hess,​ Erik P.,​Knoedler,​ Meghan A.,​Shah,​ Nilay D.,​Kline,​ Jeffrey A.,​Breslin,​ Maggie,​Branda,​ Megan E.,​Pencille,​ Laurie J.,​Asplin,​ Brent R.,​Nestler,​ David M.,​Sadosty,​ Annie T.,​Stiell,​ Ian G.,​Ting,​ Henry H.,​Montori,​ Victor M.**. The chest pain choice decision aid: a randomized trial. *Circulation. Cardiovascular quality and outcomes.* 2012. 5:251-9 |
| **Hess EP, Hollander JE, Schaffer JT, et al.** Shared decision making in patients with low risk chest pain: prospective randomized pragmatic trial. *BMJ*. 2016;355:i6165. Published 2016 Dec 5. doi:10.1136/bmj.i6165 |
| **Hopkins,​ Ria E.,​Bui,​ Thuy,​Konstantatos,​ Alex H.,​Arnold,​ Carolyn,​Magliano,​ Dianna J.,​Liew,​ Danny,​Dooley,​ Michael J.**. Educating junior doctors and pharmacists to reduce discharge prescribing of opioids for surgical patients: a cluster randomised controlled trial. *The Medical journal of Australia.* 2020. 213:417-423 |
| **Hrisos,​ S.,​Eccles,​ M.,​Johnston,​ M.,​Francis,​ J.,​Kaner,​ E. F.,​Steen,​ N.,​Grimshaw,​ J.**. An intervention modelling experiment to change GPs' intentions to implement evidence-based practice: using theory-based interventions to promote GP management of upper respiratory tract infection without prescribing antibiotics #2. *BMC Health Serv Res.* 2008. 8:10 |
| **Huizing,​ A. R.,​Hamers,​ J. P.,​Gulpers,​ M. J.,​Berger,​ M. P.**. Preventing the use of physical restraints on residents newly admitted to psycho-geriatric nursing home wards: a cluster-randomized trial. *Int J Nurs Stud.* 2009. 46:459-69 |
| **Huizing,​ A. R.,​Hamers,​ J. P.,​Gulpers,​ M. J.,​Berger,​ M. P.**. A cluster-randomized trial of an educational intervention to reduce the use of physical restraints with psychogeriatric nursing home residents. *J Am Geriatr Soc.* 2009. 57:1139-48 |
| **Hurlimann,​ David,​Limacher,​ Andreas,​Schabel,​ Maria,​Zanetti,​ Giorgio,​Berger,​ Christoph,​Muhlemann,​ Kathrin,​Kronenberg,​ Andreas,​Swiss Sentinel Working,​ Group**. Improvement of antibiotic prescription in outpatient care: a cluster-randomized intervention study using a sentinel surveillance network of physicians. *The Journal of antimicrobial chemotherapy.* 2015. 70:602-8 |
| **Ilett,​ K. F.,​Johnson,​ S.,​Greenhill,​ G.,​Mullen,​ L.,​Brockis,​ J.,​Golledge,​ C. L.,​Reid,​ D. B.**. Modification of general practitioner prescribing of antibiotics by use of a therapeutics adviser (academic detailer). *British journal of clinical pharmacology.* 2000. 49:168-73 |
| **Jarvik,​ Jeffrey G.,​Meier,​ Eric N.,​James,​ Kathryn T.,​Gold,​ Laura S.,​Tan,​ Katherine W.,​Kessler,​ Larry G.,​Suri,​ Pradeep,​Kallmes,​ David F.,​Cherkin,​ Daniel C.,​Deyo,​ Richard A.,​Sherman,​ Karen J.,​Halabi,​ Safwan S.,​Comstock,​ Bryan A.,​Luetmer,​ Patrick H.,​Avins,​ Andrew L.,​Rundell,​ Sean D.,​Griffith,​ Brent,​Friedly,​ Janna L.,​Lavallee,​ Danielle C.,​Stephens,​ Kari A.,​Turner,​ Judith A.,​Bresnahan,​ Brian W.,​Heagerty,​ Patrick J.**. The Effect of Including Benchmark Prevalence Data of Common Imaging Findings in Spine Image Reports on Health Care Utilization Among Adults Undergoing Spine Imaging: A Stepped-Wedge Randomized Clinical Trial. *JAMA network open.* 2020. 3:e2015713 |
| **Jenkins,​ Timothy C.,​Irwin,​ Amy,​Coombs,​ Letoynia,​Dealleaume,​ Lauren,​Ross,​ Stephen E.,​Rozwadowski,​ Jeanne,​Webster,​ Brian,​Dickinson,​ L. Miriam,​Sabel,​ Allison L.,​Mackenzie,​ Thomas D.,​West,​ David R.,​Price,​ Connie S.**. Effects of clinical pathways for common outpatient infections on antibiotic prescribing. *The American journal of medicine.* 2013. 126:327-335.e12 |
| **Kaboré,​ C.,​Ridde,​ V.,​Chaillet,​ N.,​Yaya Bocoum,​ F.,​Betrán,​ A. P.,​Dumont,​ A.**. DECIDE: A cluster-randomized controlled trial to reduce unnecessary caesarean deliveries in Burkina Faso. *BMC Medicine.* 2019. 17:#pages# |
| **Kerfoot,​ B. P.,​Lawler,​ E. V.,​Sokolovskaya,​ G.,​Gagnon,​ D.,​Conlin,​ P. R.**. Durable improvements in prostate cancer screening from online spaced education: A randomized controlled trial. *American Journal of Preventive Medicine.* 2010. 39:472-478 |
| **Kerry,​ S.,​Oakeshott,​ P.,​Dundas,​ D.,​Williams,​ J.**. Influence of postal distribution of the Royal College of Radiologists' guidelines,​ together with feedback on radiological referral rates,​ on X-ray referrals from general practice: a randomized controlled trial. *Family practice.* 2000. 17:46-52 |
| **Khalil,​ G. M.,​Alghasham,​ A. A.,​Abdelraheem,​ Y. F.**. Effect of establishment of treatment guidelines on antibiotic prescription pattern for children with upper respiratory tract infection. *Life Science Journal.* 2012. 9:481-486 |
| **Khan,​ A. I.,​ Mack,​ J. A.,​ Salimuzzaman,​ M.,​ Zion,​ M. I.,​ Sujon,​ H.,​ Ball,​ R. L.,​ Maples,​ S.,​ Rashid,​ M. M.,​ Chisti,​ M. J.,​ Sarker,​ S. A.,​ Biswas,​ D.,​ Hossin,​ R.,​ Bardosh,​ K. L.,​ Begum,​ Y. A.,​ Ahmed,​ A.,​ Pieri,​ D.,​ Haque,​ F.,​ Rahman,​ M.,​ Levine,​ A. C.,​ Qadri,​ F.,​ Flora,​ M. S.,​ Gurka,​ M. J.,​ Nelson,​ E. J.**. Electronic decision support and diarrhoeal disease guideline adherence (mHDM): a cluster randomised controlled trial. *The Lancet Digital Health.* 2020. 2:e250-e258 |
| **Kline,​ Jeffrey A.,​Zeitouni,​ Raghid A.,​Hernandez-Nino,​ Jackeline,​Jones,​ Alan E.**. Randomized trial of computerized quantitative pretest probability in low-risk chest pain patients: effect on safety and resource use. *Annals of emergency medicine.* 2009. 53:727-35.e1 |
| **Koczy,​ P.,​Becker,​ C.,​Rapp,​ K.,​Klie,​ T.,​Beische,​ D.,​Büchele,​ G.,​Kleiner,​ A.,​Guerra,​ V.,​Rissmann,​ U.,​Kurrle,​ S.,​Bredthauer,​ D.**. Effectiveness of a multifactorial intervention to reduce physical restraints in nursing home residents. *J Am Geriatr Soc.* 2011. 59:333-9 |
| **Kop̈ke,​ S.,​Muḧlhauser,​ I.,​Gerlach,​ A.,​Haut,​ A.,​Haastert,​ B.,​Moḧler,​ R.,​Meyer,​ G.**. Effect of a guideline-based multicomponent intervention on use of physical restraints in nursing homes: A randomized controlled trial. *JAMA - Journal of the American Medical Association.* 2012. 307:2177-2184 |
| **Kronman,​ Matthew P.,​ Gerber,​ Jeffrey S.,​ Grundmeier,​ Robert W.,​ Zhou,​ Chuan,​ Robinson,​ Jeffrey D.,​ Heritage,​ John,​ Stout,​ James,​ Burges,​ Dennis,​ Hedrick,​ Benjamin,​ Warren,​ Louise,​ Shalowitz,​ Madeleine,​ Shone,​ Laura P.,​ Steffes,​ Jennifer,​ Wright,​ Margaret,​ Fiks,​ Alexander G.,​ Mangione-Smith,​ Rita**. Reducing Antibiotic Prescribing in Primary Care for Respiratory Illness. *Pediatrics.* 2020. #volume#:#pages# |
| **Kullgren,​ Jeffrey Todd,​Krupka,​ Erin,​Schachter,​ Abigail,​Linden,​ Ariel,​Miller,​ Jacquelyn,​Acharya,​ Yubraj,​Alford,​ James,​Duffy,​ Richard,​Adler-Milstein,​ Julia**. Precommitting to choose wisely about low-value services: a stepped wedge cluster randomised trial. *BMJ quality & safety.* 2018. 27:355-364 |
| **Lampen-Smith,​ A.,​Young,​ J.,​O'Rourke,​ M. A.,​Balram,​ A.,​Inns,​ S.**. Blinded randomised controlled study of the effect of a discharge communication template on proton pump inhibitor prescribing. *New Zealand Medical Journal.* 2012. 125:#pages# |
| **Le Corvoisier,​ Philippe,​Renard,​ Vincent,​Roudot-Thoraval,​ Francoise,​Cazalens,​ Thierry,​Veerabudun,​ Kalaivani,​Canoui-Poitrine,​ Florence,​Montagne,​ Olivier,​Attali,​ Claude**. Long-term effects of an educational seminar on antibiotic prescribing by GPs: a randomised controlled trial. *The British journal of general practice : the journal of the Royal College of General Practitioners.* 2013. 63:e455-64  **Ferrat,​ E.,​Le Breton,​ J.,​Guery,​ E.,​Adeline,​ F.,​Audureau,​ E.,​Montagne,​ O.,​Roudot-Thoraval,​ F.,​Attali,​ C.,​Le Corvoisier,​ P.,​Renard,​ V.**. Effects 4.5 years after an interactive GP educational seminar on antibiotic therapy for respiratory tract infections: a randomized controlled trial. *Family practice.* 2016. 33:192-9 |
| **Lee,​ Magdalene Hui Min,​Pan,​ Darius Shaw Teng,​Huang,​ Joyce Huixin,​Chen,​ Mark I. Cheng,​Chong,​ Joash Wen Chen,​Goh,​ Ee Hui,​Jiang,​ Lili,​Leo,​ Yee Sin,​Lee,​ Tau Hong,​Wong,​ Chia Siong,​Loh,​ Victor Weng Keong,​Lim,​ Fong Seng,​Poh,​ Adrian Zhongxian,​Tham,​ Tat Yean,​Wong,​ Wei Mon,​Yu,​ Yue**. Results from a Patient-Based Health Education Intervention in Reducing Antibiotic Use for Acute Upper Respiratory Tract Infections in the Private Sector Primary Care Setting in Singapore. *Antimicrobial agents and chemotherapy.* 2017. 61:#pages# |
| **Legare,​ France,​Labrecque,​ Michel,​Cauchon,​ Michel,​Castel,​ Josette,​Turcotte,​ Stephane,​Grimshaw,​ Jeremy**. Training family physicians in shared decision-making to reduce the overuse of antibiotics in acute respiratory infections: a cluster randomized trial. *CMAJ : Canadian Medical Association journal = journal de l'Association medicale canadienne.* 2012. 184:E726-34  **Légaré,​ F.,​Labrecque,​ M.,​LeBlanc,​ A.,​Njoya,​ M.,​Laurier,​ C.,​Côté,​ L.,​Godin,​ G.,​Thivierge,​ R. L.,​O'Connor,​ A.,​St-Jacques,​ S.**. Training family physicians in shared decision making for the use of antibiotics for acute respiratory infections: a pilot clustered randomized controlled trial. *Health Expect.* 2011. 14 Suppl 1:96-110 |
| **Lemiengre,​ Marieke B.,​Verbakel,​ Jan Y.,​Colman,​ Roos,​De Burghgraeve,​ Tine,​Buntinx,​ Frank,​Aertgeerts,​ Bert,​De Baets,​ Frans,​De Sutter,​ An**. Reducing inappropriate antibiotic prescribing for children in primary care: a cluster randomised controlled trial of two interventions. *The British journal of general practice : the journal of the Royal College of General Practitioners.* 2018. 68:e204-e210 |
| **Linder,​ Jeffrey A.,​Schnipper,​ Jeffrey L.,​Tsurikova,​ Ruslana,​Yu,​ D. Tony,​Volk,​ Lynn A.,​Melnikas,​ Andrea J.,​Palchuk,​ Matvey B.,​Olsha-Yehiav,​ Maya,​Middleton,​ Blackford**. Electronic health record feedback to improve antibiotic prescribing for acute respiratory infections. *The American journal of managed care.* 2010. 16:e311-9 |
| **Linder,​ Jeffrey A.,​Schnipper,​ Jeffrey L.,​Tsurikova,​ Ruslana,​Yu,​ Tony,​Volk,​ Lynn A.,​Melnikas,​ Andrea J.,​Palchuk,​ Matvey B.,​Olsha-Yehiav,​ Maya,​Middleton,​ Blackford**. Documentation-based clinical decision support to improve antibiotic prescribing for acute respiratory infections in primary care: a cluster randomised controlled trial. *Informatics in primary care.* 2009. 17:231-40 |
| **Lionis,​ C.,​Petelos,​ E.,​Shea,​ S.,​Bagiartaki,​ G.,​Tsiligianni,​ I. G.,​Kamekis,​ A.,​Tsiantou,​ V.,​Papadakaki,​ M.,​Tatsioni,​ A.,​Moschandreas,​ J.,​Saridaki,​ A.,​Bertsias,​ A.,​Faresjö,​ T.,​Faresjö,​ A.,​Martinez,​ L.,​Agius,​ D.,​Uncu,​ Y.,​Samoutis,​ G.,​Vlcek,​ J.,​Abasaeed,​ A.,​Merkouris,​ B.**. Irrational prescribing of over-the-counter (OTC) medicines in general practice: Testing the feasibility of an educational intervention among physicians in five European countries. *BMC Family Practice.* 2014. 15:#pages# |
| **Little,​ Paul,​Stuart,​ Beth,​Francis,​ Nick,​Douglas,​ Elaine,​Tonkin-Crine,​ Sarah,​Anthierens,​ Sibyl,​Cals,​ Jochen W. L.,​Melbye,​ Hasse,​Santer,​ Miriam,​Moore,​ Michael,​Coenen,​ Samuel,​Butler,​ Chris,​Hood,​ Kerenza,​Kelly,​ Mark,​Godycki-Cwirko,​ Maciek,​Mierzecki,​ Artur,​Torres,​ Antoni,​Llor,​ Carl,​Davies,​ Melanie,​Mullee,​ Mark,​O'Reilly,​ Gilly,​van der Velden,​ Alike,​Geraghty,​ Adam W. A.,​Goossens,​ Herman,​Verheij,​ Theo,​Yardley,​ Lucy,​Grace consortium**. Effects of internet-based training on antibiotic prescribing rates for acute respiratory-tract infections: a multinational,​ cluster,​ randomised,​ factorial,​ controlled trial. *Lancet (London,​ England).* 2013. 382:1175-82  **Little,​ Paul,​Stuart,​ Beth,​Francis,​ Nick,​Douglas,​ Elaine,​Tonkin-Crine,​ Sarah,​Anthierens,​ Sibyl,​Cals,​ Jochen W. L.,​Melbye,​ Hasse,​Santer,​ Miriam,​Moore,​ Michael,​Coenen,​ Samuel,​Butler,​ Chris C.,​Hood,​ Kerenza,​Kelson,​ Mark,​Godycki-Cwirko,​ Maciek,​Mierzecki,​ Artur,​Torres,​ Antoni,​Llor,​ Carl,​Davies,​ Melanie,​Mullee,​ Mark,​O'Reilly,​ Gilly,​van der Velden,​ Alike,​Geraghty,​ Adam W. A.,​Goossens,​ Herman,​Verheij,​ Theo,​Yardley,​ Lucy,​Grace consortium**. Antibiotic Prescribing for Acute Respiratory Tract Infections 12 Months After Communication and CRP Training: A Randomized Trial. *Annals of family medicine.* 2019. 17:125-132  **Oppong, Raymond,Smith, Richard D.,Little, Paul,Verheij, Theo,Butler, Christopher C.,Goossens, Herman,Coenen, Samuel,Jowett, Sue,Roberts, Tracy E.,Achana, Felix,Stuart, Beth,Coast, Joanna**. Cost-effectiveness of internet-based training for primary care clinicians on antibiotic prescribing for acute respiratory tract infections in Europe. *The Journal of antimicrobial chemotherapy.* 2018. 73:3189-3198 |
| **Liu,​ Chenxi,​Zhang,​ Xinping,​Wang,​ Xuan,​Zhang,​ Xiaopeng,​Wan,​ Jie,​Zhong,​ Fangying**. Does public reporting influence antibiotic and injection prescribing to all patients? A cluster-randomized matched-pair trial in china. *Medicine.* 2016. 95:e3965 |
| **Loeb,​ Mark,​Brazil,​ Kevin,​Lohfeld,​ Lynne,​McGeer,​ Allison,​Simor,​ Andrew,​Stevenson,​ Kurt,​Zoutman,​ Dick,​Smith,​ Stephanie,​Liu,​ Xiwu,​Walter,​ Stephen D.**. Effect of a multifaceted intervention on number of antimicrobial prescriptions for suspected urinary tract infections in residents of nursing homes: cluster randomised controlled trial. *BMJ (Clinical research ed.).* 2005. 331:669 |
| **Loeb,​ Mark,​Hunt,​ Derek,​O'Halloran,​ Kelly,​Carusone,​ Soo Chan,​Dafoe,​ Nancy,​Walter,​ Stephen D.**. Stop orders to reduce inappropriate urinary catheterization in hospitalized patients: a randomized controlled trial. *Journal of general internal medicine.* 2008. 23:816-20 |
| **Lomas J, Enkin M, Anderson GM, Hannah WJ, Vayda E, Singer J.** Opinion leaders vs audit and feedback to implement practice guidelines. Delivery after previous cesarean section. *JAMA*. 1991;265(17):2202-2207. |
| **Macfarlane,​ John,​Holmes,​ William,​Gard,​ Philip,​Thornhill,​ David,​Macfarlane,​ Rosamund,​Hubbard,​ Richard**. Reducing antibiotic use for acute bronchitis in primary care: blinded,​ randomised controlled trial of patient information leaflet. *BMJ (Clinical research ed.).* 2002. 324:91-4 |
| **Mainous Iii,​ A. G.,​Hueston,​ W. J.,​Love,​ M. M.,​Evans,​ M. E.,​Finger,​ R.**. An evaluation of statewide strategies to reduce antibiotic overuse. *Family Medicine.* 2000. 32:22-29 |
| **Mann,​ Devin,​Hess,​ Rachel,​McGinn,​ Thomas,​Richardson,​ Safiya,​Jones,​ Simon,​Palmisano,​ Joseph,​Chokshi,​ Sara Kuppin,​Mishuris,​ Rebecca,​McCullagh,​ Lauren,​Park,​ Linda,​Dinh-Le,​ Catherine,​Smith,​ Paul,​Feldstein,​ David**. Impact of Clinical Decision Support on Antibiotic Prescribing for Acute Respiratory Infections: a Cluster Randomized Implementation Trial. *Journal of general internal medicine.* 2020. 35:788-795 |
| **Martins,​ Carlos Manuel Silva,​da Costa Teixeira,​ Andreia Sofia,​de Azevedo,​ Luis Filipe Ribeiro,​Sa,​ Luisa Maria Barbosa,​Santos,​ Paulo Alexandre Azevedo Pereira,​do Couto,​ Maria Luciana Gomes Domingues,​da Costa Pereira,​ Altamiro Manuel Rodrigues,​Hespanhol,​ Alberto Augusto Oliveira Pinto,​da Costa Santos,​ Cristina Maria Nogueira**. The effect of a test ordering software intervention on the prescription of unnecessary laboratory tests - a randomized controlled trial. *BMC medical informatics and decision making.* 2017. 17:20 |
| **Masiá,​ M.,​Matoses,​ C.,​Padilla,​ S.,​Murcia,​ A.,​Sánchez,​ V.,​Romero,​ I.,​Navarro,​ A.,​Hernández,​ I.,​Gutiérrez,​ F.**. Limited efficacy of a nonrestricted intervention on antimicrobial prescription of commonly used antibiotics in the hospital setting: Results of a randomized controlled trial. *European Journal of Clinical Microbiology and Infectious Diseases.* 2008. 27:597-605 |
| **McGinn,​ Thomas G.,​McCullagh,​ Lauren,​Kannry,​ Joseph,​Knaus,​ Megan,​Sofianou,​ Anastasia,​Wisnivesky,​ Juan P.,​Mann,​ Devin M.**. Efficacy of an evidence-based clinical decision support in primary care practices: a randomized clinical trial. *JAMA internal medicine.* 2013. 173:1584-91 |
| **McIsaac,​ W. J.,​Goel,​ V.**. Effect of an explicit decision-support tool on decisions to prescribe antibiotics for sore throat. *Medical decision making : an international journal of the Society for Medical Decision Making.* 1998. 18:220-8 |
| **McIsaac,​ Warren J.,​Goel,​ Vivek,​To,​ Teresa,​Permaul,​ Joanne A.,​Low,​ Donald E.**. Effect on antibiotic prescribing of repeated clinical prompts to use a sore throat score: lessons from a failed community intervention study. *The Journal of family practice.* 2002. 51:339-44 |
| **McNulty,​ Cliodna,​Hawking,​ Meredith,​Lecky,​ Donna,​Jones,​ Leah,​Owens,​ Rebecca,​Charlett,​ Andre,​Butler,​ Chris,​Moore,​ Philippa,​Francis,​ Nick**. Effects of primary care antimicrobial stewardship outreach on antibiotic use by general practice staff: pragmatic randomized controlled trial of the TARGET antibiotics workshop. *The Journal of antimicrobial chemotherapy.* 2018. 73:1423-1432 |
| **Meeker,​ Daniella,​Knight,​ Tara K.,​Friedberg,​ Mark W.,​Linder,​ Jeffrey A.,​Goldstein,​ Noah J.,​Fox,​ Craig R.,​Rothfeld,​ Alan,​Diaz,​ Guillermo,​Doctor,​ Jason N.**. Nudging guideline-concordant antibiotic prescribing: a randomized clinical trial. *JAMA internal medicine.* 2014. 174:425-31 |
| **Meeker,​ Daniella,​Linder,​ Jeffrey A.,​Fox,​ Craig R.,​Friedberg,​ Mark W.,​Persell,​ Stephen D.,​Goldstein,​ Noah J.,​Knight,​ Tara K.,​Hay,​ Joel W.,​Doctor,​ Jason N.**. Effect of Behavioral Interventions on Inappropriate Antibiotic Prescribing Among Primary Care Practices: A Randomized Clinical Trial. *JAMA.* 2016. 315:562-70  **Linder, Jeffrey A.,Meeker, Daniella,Fox, Craig R.,Friedberg, Mark W.,Persell, Stephen D.,Goldstein, Noah J.,Doctor, Jason N.**. Effects of Behavioral Interventions on Inappropriate Antibiotic Prescribing in Primary Care 12 Months After Stopping Interventions. *JAMA.* 2017. 318:1391-1392 |
| **Metlay,​ Joshua P.,​Camargo,​ Carlos A.,​ Jr.,​MacKenzie,​ Thomas,​McCulloch,​ Charles,​Maselli,​ Judith,​Levin,​ Sara K.,​Kersey,​ Ayanna,​Gonzales,​ Ralph,​Impaact Investigators**. Cluster-randomized trial to improve antibiotic use for adults with acute respiratory infections treated in emergency departments. *Annals of emergency medicine.* 2007. 50:221-30 |
| **Michael,​ Sean S.,​Babu,​ Kavita M.,​Androski,​ Christopher,​ Jr.,​Reznek,​ Martin A.**. Effect of a Data-driven Intervention on Opioid Prescribing Intensity Among Emergency Department Providers: A Randomized Controlled Trial. *Academic emergency medicine : official journal of the Society for Academic Emergency Medicine.* 2018. 25:482-493 |
| **Midlöv,​ Patrik,​Bondesson,​** . Effects of educational outreach visits on prescribing of benzodiazepines and antipsychotic drugs to elderly patients in primary health care in southern Sweden. *Family Practice.* 2005. 23:60-64 |
| **Milos,​ Veronica,​Jakobsson,​ Ulf,​Westerlund,​ Tommy,​Melander,​ Eva,​Molstad,​ Sigvard,​Midlov,​ Patrik**. Theory-based interventions to reduce prescription of antibiotics--a randomized controlled trial in Sweden. *Family practice.* 2013. 30:634-40 |
| **Molina López,​ T.,​Domínguez Camacho,​ J. C.,​Santos Lozano,​ J. M.,​Carbonell Carrillo,​ A.,​Sánchez Acevedo,​ J.,​Paz León,​ M. L.**. Efficacy of educational sessions to modify the prescription of new drugs. *Atencion Primaria.* 2005. 36:367-372 |
| **Monette,​ Johanne,​Miller,​ Mark A.,​Monette,​ Michele,​Laurier,​ Claudine,​Boivin,​ Jean-Francois,​Sourial,​ Nadia,​Le Cruguel,​ Jean-Pierre,​Vandal,​ Alain,​Cotton-Montpetit,​ Marie**. Effect of an educational intervention on optimizing antibiotic prescribing in long-term care facilities. *Journal of the American Geriatrics Society.* 2007. 55:1231-5 |
| **Montoy,​ Juan Carlos C.,​Coralic,​ Zlatan,​Herring,​ Andrew A.,​Clattenburg,​ Eben J.,​Raven,​ Maria C.**. Association of Default Electronic Medical Record Settings With Health Care Professional Patterns of Opioid Prescribing in Emergency Departments: A Randomized Quality Improvement Study. *JAMA internal medicine.* 2020. 180:487-493 |
| **Nace,​ David A.,​ Hanlon,​ Joseph T.,​ Crnich,​ Christopher J.,​ Drinka,​ Paul J.,​ Schweon,​ Steven J.,​ Anderson,​ Gulsum,​ Perera,​ Subashan**. A Multifaceted Antimicrobial Stewardship Program for the Treatment of Uncomplicated Cystitis in Nursing Home Residents. *JAMA internal medicine.* 2020.  **Hanlon,​ Joseph T.,​Perera,​ Subashan,​Schweon,​ Steven,​Drinka,​ Paul,​Crnich,​ Christopher,​Nace,​ David A.**. Improvements in Antibiotic Appropriateness for Cystitis in Older Nursing Home Residents: A Quality Improvement Study With Randomized Assignment. *Journal of the American Medical Directors Association.* 2021. 22:173-177 |
| **Najafi,​ Nader,​Cucina,​ Russ,​Pierre,​ Bruce,​Khanna,​ Raman**. Assessment of a Targeted Electronic Health Record Intervention to Reduce Telemetry Duration: A Cluster-Randomized Clinical Trial. *JAMA internal medicine.* 2019. 179:11-15 |
| **Naughton,​ Corina,​Feely,​ John,​Bennett,​ Kathleen**. A RCT evaluating the effectiveness and cost-effectiveness of academic detailing versus postal prescribing feedback in changing GP antibiotic prescribing. *Journal of evaluation in clinical practice.* 2009. 15:807-12 |
| **Nejad,​ Afshin Sarafi,​Noori,​ Mohammad Reza Farrokhi,​Haghdoost,​ Ali Akbar,​Bahaadinbeigy,​ Kambiz,​Abu-Hanna,​ Ameen,​Eslami,​ Saeid**. The effect of registry-based performance feedback via short text messages and traditional postal letters on prescribing parenteral steroids by general practitioners--A randomized controlled trial. *International journal of medical informatics.* 2016. 87:36-43 |
| **Neven,​ Darin,​Paulozzi,​ Leonard,​Howell,​ Donelle,​McPherson,​ Sterling,​Murphy,​ Sean M.,​Grohs,​ Becky,​Marsh,​ Linda,​Lederhos,​ Crystal,​Roll,​ John**. A Randomized Controlled Trial of a Citywide Emergency Department Care Coordination Program to Reduce Prescription Opioid Related Emergency Department Visits. *The Journal of emergency medicine.* 2016. 51:498-507  **Murphy, Sean M.,Howell, Donelle,McPherson, Sterling,Grohs, Rebecca,Roll, John,Neven, Darin**. A Randomized Controlled Trial of a Citywide Emergency Department Care-Coordination Program to Reduce Prescription Opioid-Related Visits: An Economic Evaluation. *The Journal of emergency medicine.* 2017. 53:186-194 |
| **Ngasala,​ Billy,​Mubi,​ Marycelina,​Warsame,​ Marian,​Petzold,​ Max G.,​Massele,​ Amos Y.,​Gustafsson,​ Lars L.,​Tomson,​ Goran,​Premji,​ Zul,​Bjorkman,​ Anders**. Impact of training in clinical and microscopy diagnosis of childhood malaria on antimalarial drug prescription and health outcome at primary health care level in Tanzania: a randomized controlled trial. *Malaria journal.* 2008. 7:199 |
| **Nikolajevic-Sarunac,​ J.,​Henry,​ D. A.,​O'Connell,​ D. L.,​Robertson,​ J.**. Effects of information framing on the intentions of family physicians to prescribe long-term hormone replacement therapy. *Journal of general internal medicine.* 1999. 14:591-8 |
| **Nsimba,​ S. E. D.,​Massele,​ A.,​Kayombo,​ E. J.**. Reducing injection overuse through consumers'-prescribers' interactional group discussions in Dar es Salaam region,​ Tanzania. *Therapy.* 2011. 8:179-187 |
| **O'Connell DL, Henry D, Tomlins R.** Randomised controlled trial of effect of feedback on general practitioners' prescribing in Australia. *BMJ*. 1999;318(7182):507-511. doi:10.1136/bmj.318.7182.507 |
| **Pagaiya,​ N.,​Garner,​ P.**. Primary care nurses using guidelines in Thailand: a randomized controlled trial. *Trop Med Int Health.* 2005. 10:471-7 |
| **Palen,​ Ted E.,​ Sharpe,​ Richard E.,​ Jr.,​ Shetterly,​ Susan M.,​ Steiner,​ John F.**. Randomized Clinical Trial of a Clinical Decision Support Tool for Improving the Appropriateness Scores for Ordering Imaging Studies in Primary and Specialty Care Ambulatory Clinics. *AJR. American journal of roentgenology.* 2019. 213:1015-1020 |
| **Pasay,​ Darren K.,​ Guirguis,​ Micheal S.,​ Shkrobot,​ Rhonda C.,​ Slobodan,​ Jeremy P.,​ Wagg,​ Adrian S.,​ Sadowski,​ Cheryl A.,​ Conly,​ John M.,​ Saxinger,​ Lynora M.,​ Bresee,​ Lauren C.**. Antimicrobial stewardship in rural nursing homes: Impact of interprofessional education and clinical decision tool implementation on urinary tract infection treatment in a cluster randomized trial. *Infection control and hospital epidemiology.* 2019. 40:432-437 |
| **Pellfolk,​ T. J.,​Gustafson,​ Y.,​Bucht,​ G.,​Karlsson,​ S.**. Effects of a restraint minimization program on staff knowledge,​ attitudes,​ and practice: a cluster randomized trial. *J Am Geriatr Soc.* 2010. 58:62-9 |
| **Persell,​ Stephen D.,​Doctor,​ Jason N.,​Friedberg,​ Mark W.,​Meeker,​ Daniella,​Friesema,​ Elisha,​Cooper,​ Andrew,​Haryani,​ Ajay,​Gregory,​ Dyanna L.,​Fox,​ Craig R.,​Goldstein,​ Noah J.,​Linder,​ Jeffrey A.**. Behavioral interventions to reduce inappropriate antibiotic prescribing: a randomized pilot trial. *BMC infectious diseases.* 2016. 16:373 |
| **Pettersson,​ Eva,​Vernby,​ Asa,​Molstad,​ Sigvard,​Lundborg,​ Cecilia Stalsby**. Can a multifaceted educational intervention targeting both nurses and physicians change the prescribing of antibiotics to nursing home residents? A cluster randomized controlled trial. *The Journal of antimicrobial chemotherapy.* 2011. 66:2659-66 |
| **Phuong,​ Hoang L.,​Nga,​ Tran T. T.,​Giao,​ Phan T.,​Hung,​ Le Q.,​Binh,​ Tran Q.,​Nam,​ Nguyen V.,​Nagelkerke,​ Nico,​de Vries,​ Peter J.**. Randomised primary health center based interventions to improve the diagnosis and treatment of undifferentiated fever and dengue in Vietnam. *BMC health services research.* 2010. 10:275 |
| **Pimlott,​ Nicholas J. G.,​Hux,​ Janet E.,​Wilson,​ Lynn M.,​Kahan,​ Meldon,​Li,​ Cindy,​Rosser,​ Walter W.**. Educating physicians to reduce benzodiazepine use by elderly patients: a randomized controlled trial. *CMAJ : Canadian Medical Association journal = journal de l'Association medicale canadienne.* 2003. 168:835-9 |
| **Pinto,​ D.,​Heleno,​ B.,​Rodrigues,​ D. S.,​Papoila,​ A. L.,​Santos,​ I.,​Caetano,​ P. A.**. Effectiveness of educational outreach visits compared with usual guideline dissemination to improve family physician prescribing-an 18-month open cluster-randomized trial. *Implementation Science.* 2018. 13:#pages# |
| **Pshetizky,​ Y.,​Naimer,​ S.,​Shvartzman,​ P.**. Acute otitis media--a brief explanation to parents and antibiotic use. *Fam Pract.* 2003. 20:417-9 |
| **Raebel,​ M. A.,​Charles,​ J.,​Dugan,​ J.,​Carroll,​ N. M.,​Korner,​ E. J.,​Brand,​ D. W.,​Magid,​ D. J.**. Randomized trial to improve prescribing safety in ambulatory elderly patients. *Journal of the American Geriatrics Society.* 2007. 55:977-985 |
| **Rambaud-Althaus,​ Clotilde,​Shao,​ Amani,​Samaka,​ Josephine,​Swai,​ Ndeniria,​Perri,​ Seneca,​Kahama-Maro,​ Judith,​Mitchell,​ Marc,​D'Acremont,​ Valerie,​Genton,​ Blaise**. Performance of Health Workers Using an Electronic Algorithm for the Management of Childhood Illness in Tanzania: A Pilot Implementation Study. *The American journal of tropical medicine and hygiene.* 2017. 96:249-257 |
| **Ray,​ W. A.,​Stein,​ C. M.,​Byrd,​ V.,​Shorr,​ R.,​Pichert,​ J. W.,​Gideon,​ P.,​Arnold,​ K.,​Brandt,​ K. D.,​Pincus,​ T.,​Griffin,​ M. R.**. Educational program for physicians to reduce use of non-steroidal anti-inflammatory drugs among community-dwelling elderly persons: a randomized controlled trial. *Medical care.* 2001. 39:425-35 |
| **Regev-Yochay,​ Gili,​Raz,​ Meir,​Dagan,​ Ron,​Roizin,​ Hector,​Morag,​ Benjamin,​Hetman,​ Shmuel,​Ringel,​ Sigal,​Ben-Israel,​ Neta,​Varon,​ Miriam,​Somekh,​ Eli,​Rubinstein,​ Ethan**. Reduction in antibiotic use following a cluster randomized controlled multifaceted intervention: the Israeli judicious antibiotic prescription study. *Clinical infectious diseases : an official publication of the Infectious Diseases Society of America.* 2011. 53:33-41 |
| **Ringwalt,​ Chris,​Shanahan,​ Meghan,​Wodarski,​ Stephanie,​Jones,​ Jennifer,​Schaffer,​ Danielle,​Fusaro,​ Angela,​Paulozzi,​ Len,​Garrettson,​ Mariana,​Ford,​ Marsha**. A Randomized Controlled Trial of an Emergency Department Intervention for Patients with Chronic Noncancer Pain. *The Journal of emergency medicine.* 2015. 49:974-83 |
| **Rognstad,​ Sture,​Brekke,​ Mette,​Fetveit,​ Arne,​Dalen,​ Ingvild,​Straand,​ Jorund**. Prescription peer academic detailing to reduce inappropriate prescribing for older patients: a cluster randomised controlled trial. *The British journal of general practice : the journal of the Royal College of General Practitioners.* 2013. 63:e554-62 |
| **Rothschild,​ Jeffrey M.,​McGurk,​ Siobhan,​Honour,​ Melissa,​Lu,​ Linh,​McClendon,​ Aubre A.,​Srivastava,​ Priya,​Churchill,​ W. Hallowell,​Kaufman,​ Richard M.,​Avorn,​ Jerry,​Cook,​ E. Francis,​Bates,​ David W.**. Assessment of education and computerized decision support interventions for improving transfusion practice. *Transfusion.* 2007. 47:228-39 |
| **Ruangkanchanasetr,​ S.**. Laboratory investigation utilization in pediatric out-patient department Ramathibodi Hospital. *Journal of the Medical Association of Thailand = Chotmaihet thangphaet.* 1993. 76 Suppl 2:194-208 |
| **Ryskina,​ Kira,​Jessica Dine,​ C.,​Gitelman,​ Yevgeniy,​Leri,​ Damien,​Patel,​ Mitesh,​Kurtzman,​ Gregory,​Lin,​ Lisa Y.,​Epstein,​ Andrew J.**. Effect of Social Comparison Feedback on Laboratory Test Ordering for Hospitalized Patients: A Randomized Controlled Trial. *Journal of general internal medicine.* 2018. 33:1639-1645 |
| **Sacarny,​ Adam,​Barnett,​ Michael L.,​Le,​ Jackson,​Tetkoski,​ Frank,​Yokum,​ David,​Agrawal,​ Shantanu**. Effect of Peer Comparison Letters for High-Volume Primary Care Prescribers of Quetiapine in Older and Disabled Adults: A Randomized Clinical Trial. *JAMA psychiatry.* 2018. 75:1003-1011 |
| **Sacarny,​ Adam,​Yokum,​ David,​Finkelstein,​ Amy,​Agrawal,​ Shantanu**. Medicare Letters To Curb Overprescribing Of Controlled Substances Had No Detectable Effect On Providers. *Health affairs (Project Hope).* 2016. 35:471-9  **Sacarny,​ Adam,​Olenski,​ Andrew R.,​Barnett,​ Michael L.**. Association of Quetiapine Overuse Letters With Prescribing by Physician Peers of Targeted Recipients: A Secondary Analysis of a Randomized Clinical Trial. *JAMA psychiatry.* 2019. 76:1094-1095 |
| **Sallis,​ Anna,​ Bondaronek,​ Paulina,​ Sanders,​ Jet G.,​ Yu,​ Ly-Mee,​ Harris,​ Victoria,​ Vlaev,​ Ivo,​ Sanders,​ Michael,​ Tonkin-Crine,​ Sarah,​ Chadborn,​ Tim**. Prescriber Commitment Posters to Increase Prudent Antibiotic Prescribing in English General Practice: A Cluster Randomized Controlled Trial. *Antibiotics (Basel,​ Switzerland).* 2020. 9:#pages# |
| **Samore,​ Matthew H.,​Bateman,​ Kim,​Alder,​ Stephen C.,​Hannah,​ Elizabeth,​Donnelly,​ Sharon,​Stoddard,​ Gregory J.,​Haddadin,​ Bassam,​Rubin,​ Michael A.,​Williamson,​ Jacquelyn,​Stults,​ Barry,​Rupper,​ Randall,​Stevenson,​ Kurt**. Clinical decision support and appropriateness of antimicrobial prescribing: a randomized trial. *JAMA.* 2005. 294:2305-14 |
| **Schectman,​ Joel M.,​Schroth,​ W. Scott,​Verme,​ Dante,​Voss,​ John D.**. Randomized controlled trial of education and feedback for implementation of guidelines for acute low back pain. *Journal of general internal medicine.* 2003. 18:773-80 |
| **Schmidt,​ I.,​Claesson,​ C. B.,​Westerholm,​ B.,​Nilsson,​ L. G.,​Svarstad,​ B. L.**. The impact of regular multidisciplinary team interventions on psychotropic prescribing in Swedish nursing homes. *Journal of the American Geriatrics Society.* 1998. 46:77-82 |
| **Schneiderman,​ L. J.,​Gilmer,​ T.,​Teetzel,​ H. D.,​Dugan,​ D. O.,​Blustein,​ J.,​Cranford,​ R.,​Briggs,​ K. B.,​Komatsu,​ G. I.,​Goodman-Crews,​ P.,​Cohn,​ F.,​Young,​ E. W.**. Effect of ethics consultations on nonbeneficial life-sustaining treatments in the intensive care setting: a randomized controlled trial. *Jama.* 2003. 290:1166-72 |
| **Sedrak,​ Mina S.,​Myers,​ Jennifer S.,​Small,​ Dylan S.,​Nachamkin,​ Irving,​Ziemba,​ Justin B.,​Murray,​ Dana,​Kurtzman,​ Gregory W.,​Zhu,​ Jingsan,​Wang,​ Wenli,​Mincarelli,​ Deborah,​Danoski,​ Daniel,​Wells,​ Brian P.,​Berns,​ Jeffrey S.,​Brennan,​ Patrick J.,​Hanson,​ C. William,​Dine,​ C. Jessica,​Patel,​ Mitesh S.**. Effect of a Price Transparency Intervention in the Electronic Health Record on Clinician Ordering of Inpatient Laboratory Tests: The PRICE Randomized Clinical Trial. *JAMA internal medicine.* 2017. 177:939-945 |
| **Sharma,​ S.,​Gupta,​ U.,​Roy Chaudhury,​ R.,​Bapna,​ J. S.**. Prescribing Behaviour of Physicians. *Journal of Health Management.* 2002. 4:55-71 |
| **Sharma,​ Sweekriti,​Traeger,​ Adrian C.,​O'Keeffe,​ Mary,​Copp,​ Tessa,​Freeman,​ Alexandra,​Hoffmann,​ Tammy,​Maher,​ Chris G.**. Effect of information format on intentions and beliefs regarding diagnostic imaging for non-specific low back pain: A randomised controlled trial in members of the public. *Patient education and counseling.* 2021. 104:595-602 |
| **Shen,​ XingRong,​Lu,​ Manman,​Feng,​ Rui,​Cheng,​ Jing,​Chai,​ Jing,​Xie,​ Maomao,​Dong,​ Xuemeng,​Jiang,​ Tao,​Wang,​ Debin**. Web-Based Just-in-Time Information and Feedback on Antibiotic Use for Village Doctors in Rural Anhui,​ China: Randomized Controlled Trial. *Journal of medical Internet research.* 2018. 20:e53 |
| **Shojania,​ K. G.,​Yokoe,​ D.,​Platt,​ R.,​Fiskio,​ J.,​Ma'luf,​ N.,​Bates,​ D. W.**. Reducing vancomycin use utilizing a computer guideline: results of a randomized controlled trial. *Journal of the American Medical Informatics Association : JAMIA.* 1998. 5:554-62 |
| **Silverberg,​ Noah D.,​Otamendi,​ Thalia,​Panenka,​ William J.,​Archambault,​ Patrick,​Babul,​ Shelina,​MacLellan,​ Anna,​Li,​ Linda C.,​Canadian,​ T. B. I. Research Consortium**. De-implementing Prolonged Rest Advice for Concussion in Primary Care Settings: A Pilot Stepped Wedge Cluster Randomized Trial. *The Journal of head trauma rehabilitation.* 2021. 36:79-86 |
| **Simon,​ Steven R.,​Smith,​ David H.,​Feldstein,​ Adrianne C.,​Perrin,​ Nancy,​Yang,​ Xiuhai,​Zhou,​ Yvonne,​Platt,​ Richard,​Soumerai,​ Stephen B.**. Computerized prescribing alerts and group academic detailing to reduce the use of potentially inappropriate medications in older people. *Journal of the American Geriatrics Society.* 2006. 54:963-8 |
| **Smith,​ Timothy D. H.,​Watt,​ Hilary,​Gunn,​ Laura,​Car,​ Josip,​Boyle,​ Robert J.**. Recommending Oral Probiotics to Reduce Winter Antibiotic Prescriptions in People With Asthma: A Pragmatic Randomized Controlled Trial. *Annals of family medicine.* 2016. 14:422-30 |
| **Solomon,​ D. H.,​Van Houten,​ L.,​Glynn,​ R. J.,​Baden,​ L.,​Curtis,​ K.,​Schrager,​ H.,​Avorn,​ J.**. Academic detailing to improve use of broad-spectrum antibiotics at an academic medical center. *Archives of internal medicine.* 2001. 161:1897-902 |
| **Sondergaard,​ Jens,​Andersen,​ Morten,​Stovring,​ Henrik,​Kragstrup,​ Jakob**. Mailed prescriber feedback in addition to a clinical guideline has no impact: a randomised,​ controlled trial. *Scandinavian journal of primary health care.* 2003. 21:47-51 |
| **Soumerai,​ S. B.,​Avorn,​ J.**. Predictors of physician prescribing change in an educational experiment to improve medication use. *Medical care.* 1987. 25:210-21 |
| **Soumerai,​ S. B.,​Salem-Schatz,​ S.,​Avorn,​ J.,​Casteris,​ C. S.,​Ross-Degnan,​ D.,​Popovsky,​ M. A.**. A controlled trial of educational outreach to improve blood transfusion practice. *JAMA.* 1993. 270:961-6 |
| **Stamm,​ Andrew W.,​Banerji,​ John S.,​Wolff,​ Erika M.,​Slee,​ April,​Akapame,​ Sydney,​Dahl,​ Kathryn,​Massman I I I,​ John D.,​Soung,​ Michael C.,​Pittenger,​ Kim R.,​Corman,​ John M.**. A decision aid versus shared decision making for prostate cancer screening: results of a randomized,​ controlled trial. *The Canadian journal of urology.* 2017. 24:8910-8917 |
| **Stein,​ C. M.,​Griffin,​ M. R.,​Taylor,​ J. A.,​Pichert,​ J. W.,​Brandt,​ K. D.,​Ray,​ W. A.**. Educational program for nursing home physicians and staff to reduce use of non-steroidal anti-inflammatory drugs among nursing home residents: a randomized controlled trial. *Medical care.* 2001. 39:436-45 |
| **Stiell,​ Ian G,​Clement,​ Catherine M,​Grimshaw,​ Jeremy,​Brison,​ Robert J,​Rowe,​ Brian H,​Schull,​ Michael J,​Lee,​ Jacques S,​Brehaut,​ Jamie,​McKnight,​ R Douglas,​Eisenhauer,​ Mary A,​Dreyer,​ Jonathan,​Letovsky,​ Eric,​Rutledge,​ Tim,​MacPhail,​ Iain,​Ross,​ Scott,​Shah,​ Amit,​Perry,​ Jeffrey J,​Holroyd,​ Brian R,​Ip,​ Urbain,​Lesiuk,​ Howard,​Wells,​ George A**. Implementation of the Canadian C-Spine Rule: prospective 12 centre cluster randomised trial. *BMJ.* 2009. 339:b4146 |
| **Suffoletto,​ Brian,​Landau,​ Aaron**. Nudging Emergency Care Providers to Reduce Opioid Prescribing Using Peer Norm Comparison Feedback: A Pilot Randomized Trial. *Pain medicine (Malden,​ Mass.).* 2019. #volume#:#pages# |
| **Tan,​ Winson Jianhong,​Acharyya,​ Sanchalika,​Chew,​ Min Hoe,​Foo,​ Fung Joon,​Chan,​ Weng Hoong,​Wong,​ Wai Keong,​Ooi,​ London Lucien,​Ng,​ Jeremy Chung Fai,​Ong,​ Hock Soo**. Randomized control trial comparing an Alvarado Score-based management algorithm and current best practice in the evaluation of suspected appendicitis. *World journal of emergency surgery : WJES.* 2020. 15:30 |
| **Tang,​ Yuqing,​Liu,​ Chaojie,​Zhang,​ Xinping**. Public reporting as a prescriptions quality improvement measure in primary care settings in China: variations in effects associated with diagnoses. *Scientific reports.* 2016. 6:39361 |
| **Tang,​ Yuqing,​Liu,​ Chenxi,​Zhang,​ Xinping**. Performance associated effect variations of public reporting in promoting antibiotic prescribing practice: a cluster randomized-controlled trial in primary healthcare settings. *Primary health care research & development.* 2017. 18:482-491 |
| **Taylor,​ J. A.,​Kwan-Gett,​ T. S.,​McMahon,​ E. M.,​ Jr.**. Effectiveness of a parental educational intervention in reducing antibiotic use in children: a randomized controlled trial. *Pediatr Infect Dis J.* 2005. 24:489-93 |
| **Teixeira Rodrigues,​ Antonio,​Roque,​ Fatima,​Pineiro-Lamas,​ Maria,​Falcao,​ Amilcar,​Figueiras,​ Adolfo,​Herdeiro,​ Maria Teresa**. Effectiveness of an intervention to improve antibiotic-prescribing behaviour in primary care: a controlled,​ interrupted time-series study. *The Journal of antimicrobial chemotherapy.* 2019. 74:2788-2796 |
| **Terrell,​ Kevin M.,​Perkins,​ Anthony J.,​Dexter,​ Paul R.,​Hui,​ Siu L.,​Callahan,​ Christopher M.,​Miller,​ Douglas K.**. Computerized decision support to reduce potentially inappropriate prescribing to older emergency department patients: a randomized,​ controlled trial. *Journal of the American Geriatrics Society.* 2009. 57:1388-94 |
| **Testad I, Ballard C, Brønnick K, Aarsland D.** The effect of staff training on agitation and use of restraint in nursing home residents with dementia: a single-blind, randomized controlled trial. *J Clin Psychiatry*. 2010;71(1):80-86. doi:10.4088/JCP.09m05486oli |
| **Testad,​ I.,​Mekki,​ T. E.,​Førland,​ O.,​Øye,​ C.,​Tveit,​ E. M.,​Jacobsen,​ F.,​Kirkevold,​ Ø**. Modeling and evaluating evidence-based continuing education program in nursing home dementia care (MEDCED)--training of care home staff to reduce use of restraint in care home residents with dementia. A cluster randomized controlled trial. *Int J Geriatr Psychiatry.* 2016. 31:24-32 |
| **Thomas,​ Ruth E.,​Croal,​ Bernard Lewis,​Ramsay,​ Craig,​Eccles,​ Martin,​Grimshaw,​ Jeremy**. Effect of enhanced feedback and brief educational reminder messages on laboratory test requesting in primary care: a cluster randomised trial. *Lancet (London,​ England).* 2006. 367:1990-6 |
| **Tierney,​ W. M.,​Miller,​ M. E.,​McDonald,​ C. J.**. The effect on test ordering of informing physicians of the charges for outpatient diagnostic tests. *N Engl J Med.* 1990. 322:1499-504 |
| **Tierney,​ William M.,​McDonald,​ Clement J.,​Hui,​ Siu L.,​Martin,​ Douglas K.**. Computer Predictions of Abnormal Test Results: Effects on Outpatient Testing. *JAMA.* 1988. 259:1194-1198 |
| **Torrente,​ Fernando,​Bustin,​ Julian,​Triskier,​ Fabian,​Ajzenman,​ Nicolas,​Tomio,​ Ailin,​Mastai,​ Ricardo,​Lopez Boo,​ Florencia**. Effect of a Social Norm Email Feedback Program on the Unnecessary Prescription of Nimodipine in Ambulatory Care of Older Adults: A Randomized Clinical Trial. *JAMA network open.* 2020. 3:e2027082 |
| **Trietsch,​ J.,​Van Steenkiste,​ B.,​Grol,​ R.,​Winkens,​ B.,​Ulenkate,​ H.,​Metsemakers,​ J.,​Van Der Weijden,​ T.**. Effect of audit and feedback with peer review on general practitioners' prescribing and test ordering performance: A cluster-randomized controlled trial. *BMC Family Practice.* 2017. 18:#pages# |
| **Ubel,​ Peter A.,​Jepson,​ Christopher,​Baron,​ Jonathan,​Hershey,​ John C.,​Asch,​ David A.**. The influence of cost-effectiveness information on physicians' cancer screening recommendations. *Social science & medicine (1982).* 2003. 56:1727-36 |
| **Urbiztondo,​ Ines,​Bjerrum,​ Lars,​Caballero,​ Lidia,​Suarez,​ Miguel Angel,​Olinisky,​ Monica,​Cordoba,​ Gloria**. Decreasing Inappropriate Use of Antibiotics in Primary Care in Four Countries in South America-Cluster Randomized Controlled Trial. *Antibiotics (Basel,​ Switzerland).* 2017. 6:#pages# |
| **van Bokhoven,​ Marloes A.,​Koch,​ Helen,​van der Weijden,​ Trudy,​Weekers-Muyres,​ Anuska H. M.,​Bindels,​ Patrick J. E.,​Grol,​ Richard P. T. M.,​Dinant,​ Geert-Jan**. The effect of watchful waiting compared to immediate test ordering instructions on general practitioners' blood test ordering behaviour for patients with unexplained complaints; a randomized clinical trial (ISRCTN55755886). *Implementation science : IS.* 2012. 7:29 |
| **van de Maat,​ Josephine S.,​ Peeters,​ Daphne,​ Nieboer,​ Daan,​ van Wermeskerken,​ Anne-Marie,​ Smit,​ Frank J.,​ Noordzij,​ Jeroen G.,​ Tramper-Stranders,​ Gerdien,​ Driessen,​ Gertjan J. A.,​ Obihara,​ Charlie C.,​ Punt,​ Jeanine,​ van der Lei,​ Johan,​ Polinder,​ Suzanne,​ Moll,​ Henriette A.,​ Oostenbrink,​ Rianne**. Evaluation of a clinical decision rule to guide antibiotic prescription in children with suspected lower respiratory tract infection in The Netherlands: A stepped-wedge cluster randomised trial. *PLoS medicine.* 2020. 17:e1003034 |
| **van der Velden,​ A. W.,​Kuyvenhoven,​ M. M.,​Verheij,​ T. J. M.**. Improving antibiotic prescribing quality by an intervention embedded in the primary care practice accreditation: The ARTI4 randomized trial. *Journal of Antimicrobial Chemotherapy.* 2016. 71:257-263 |
| **van Eijk,​ M. E.,​Avorn,​ J.,​Porsius,​ A. J.,​de Boer,​ A.**. Reducing prescribing of highly anticholinergic antidepressants for elderly people: randomised trial of group versus individual academic detailing. *BMJ (Clinical research ed.).* 2001. 322:654-7 |
| **Verstappen,​ Wim H. J. M.,​van der Weijden,​ Trudy,​Dubois,​ Willy I.,​Smeele,​ Ivo,​Hermsen,​ Jan,​Tan,​ Frans E. S.,​Grol,​ Richard P. T. M.**. Improving test ordering in primary care: the added value of a small-group quality improvement strategy compared with classic feedback only. *Annals of family medicine.* 2004. 2:569-575 |
| **Verstappen,​ Wim H. J. M.,​van der Weijden,​ Trudy,​Sijbrandij,​ Jildou,​Smeele,​ Ivo,​Hermsen,​ Jan,​Grimshaw,​ Jeremy,​Grol,​ Richard P. T. M.**. Effect of a practice-based strategy on test ordering performance of primary care physicians: a randomized trial. *JAMA.* 2003. 289:2407-12 |
| **Vervloet,​ Marcia,​Meulepas,​ Marianne A.,​Cals,​ Jochen W. L.,​Eimers,​ Marietta,​van der Hoek,​ Lucas S.,​van Dijk,​ Liset**. Reducing antibiotic prescriptions for respiratory tract infections in family practice: results of a cluster randomized controlled trial evaluating a multifaceted peer-group-based intervention. *NPJ primary care respiratory medicine.* 2016. 26:15083 |
| **Vicentini,​ Massimo,​ Mancuso,​ Pamela,​ Giorgi Rossi,​ Paolo,​ Di Pede,​ Sara,​ Pellati,​ Morena,​ Gandolfi,​ Alberto,​ Zoboli,​ Daniela,​ Ricco,​ Daniela,​ Busani,​ Corrado,​ Ferretti,​ Alessandra**. A cluster randomized trial to measure the impact on nonsteroidal anti-inflammatory drug and proton pump inhibitor prescribing in Italy of distributing cost-free paracetamol to osteoarthritic patients. *BMC family practice.* 2019. 20:169 |
| **Voorn,​ Veronique M. A.,​Marang-van de Mheen,​ Perla J.,​van der Hout,​ Anja,​Hofstede,​ Stefanie N.,​So-Osman,​ Cynthia,​van den Akker-van Marle,​ M. Elske,​Kaptein,​ Ad A.,​Stijnen,​ Theo,​Koopman-van Gemert,​ Ankie W. M. M.,​Dahan,​ Albert,​Vliet Vlieland,​ Thea P. M. M.,​Nelissen,​ Rob G. H. H.,​van Bodegom-Vos,​ Leti**. The effectiveness of a de-implementation strategy to reduce low-value blood management techniques in primary hip and knee arthroplasty: a pragmatic cluster-randomized controlled trial. *Implementation science : IS.* 2017. 12:72 |
| **Wattal,​ C.,​Goel,​ N.,​Khanna,​ S.,​Byotra,​ S. P.,​Laxminarayan,​ R.,​Easton,​ A.**. Impact of informational feedback to clinicians on antibiotic-prescribing rates in a tertiary care hospital in Delhi. *Indian journal of medical microbiology.* 2015. 33:255-9 |
| **Wei,​ Xiaolin,​Zhang,​ Zhitong,​Walley,​ John D.,​Hicks,​ Joseph P.,​Zeng,​ Jun,​Deng,​ Simin,​Zhou,​ Yu,​Yin,​ Jia,​Newell,​ James N.,​Sun,​ Qiang,​Zou,​ Guanyang,​Guo,​ Yan,​Upshur,​ Ross E. G.,​Lin,​ Mei**. Effect of a training and educational intervention for physicians and caregivers on antibiotic prescribing for upper respiratory tract infections in children at primary care facilities in rural China: a cluster-randomised controlled trial. *The Lancet. Global health.* 2017. 5:e1258-e1267  **Wei,​ Xiaolin,​Zhang,​ Zhitong,​Hicks,​ Joseph P.,​Walley,​ John D.,​King,​ Rebecca,​Newell,​ James N.,​Yin,​ Jia,​Zeng,​ Jun,​Guo,​ Yan,​Lin,​ Mei,​Upshur,​ Ross E. G.,​Sun,​ Qiang**. Long-term outcomes of an educational intervention to reduce antibiotic prescribing for childhood upper respiratory tract infections in rural China: Follow-up of a cluster-randomised controlled trial. *PLoS medicine.* 2019. 16:e1002733 |
| **Weiss,​ Curtis H.,​Dibardino,​ David,​Rho,​ Jason,​Sung,​ Nina,​Collander,​ Brett,​Wunderink,​ Richard G.**. A clinical trial comparing physician prompting with an unprompted automated electronic checklist to reduce empirical antibiotic utilization. *Critical care medicine.* 2013. 41:2563-9 |
| **Welschen,​ Ineke,​Kuyvenhoven,​ Marijke M.,​Hoes,​ Arno W.,​Verheij,​ Theo J. M.**. Effectiveness of a multiple intervention to reduce antibiotic prescribing for respiratory tract symptoms in primary care: randomised controlled trial. *BMJ (Clinical research ed.).* 2004. 329:431 |
| **Wertheim,​ Bradley M.,​Aguirre,​ Andrew J.,​Bhattacharyya,​ Roby P.,​Chorba,​ John,​Jadhav,​ Ashutosh P.,​Kerry,​ Vanessa B.,​Macklin,​ Eric A.,​Motyckova,​ Gabriela,​Raju,​ Shveta,​Lewandrowski,​ Kent,​Hunt,​ Daniel P.,​Wright,​ Douglas E.**. An Educational and Administrative Intervention to Promote Rational Laboratory Test Ordering on an Academic General Medicine Service. *The American journal of medicine.* 2017. 130:47-53 |
| **Whyte,​ John,​ Winiecki,​ Scott,​ Hoffman,​ Christina,​ Patel,​ Kaushal**. FDA collaboration to improve safe use of fluoroquinolone antibiotics: an ex post facto matched control study of targeted short-form messaging and online education served to high prescribers. *Pharmacy practice.* 2020. 18:1773 |
| **Wilson,​ Eileen J.,​Nasrin,​ Dilruba,​Dear,​ Keith B. G.,​Douglas,​ Robert M.**. Changing GPs' antibiotic prescribing: a randomised controlled trial. *Communicable diseases intelligence quarterly report.* 2003. 27 Suppl:S32-8 |
| **Wolters,​ Rene,​Wensing,​ Michel,​Klomp,​ Maarten,​Lagro-Jansen,​ Toine,​Weel,​ Chris van,​Grol,​ Richard**. Effects of distance learning on clinical management of LUTS in primary care: a randomised trial. *Patient education and counseling.* 2005. 59:212-8 |
| **Worrall,​ Graham,​Hutchinson,​ James,​Sherman,​ Gregory,​Griffiths,​ Joseph**. Diagnosing streptococcal sore throat in adults: randomized controlled trial of in-office aids. *Canadian family physician Medecin de famille canadien.* 2007. 53:666-71 |
| **Yadav,​ Kabir,​Meeker,​ Daniella,​Mistry,​ Rakesh D.,​Doctor,​ Jason N.,​Fleming-Dutra,​ Katherine E.,​Fleischman,​ Ross J.,​Gaona,​ Samuel D.,​Stahmer,​ Aubyn,​May,​ Larissa**. A Multifaceted Intervention Improves Prescribing for Acute Respiratory Infection for Adults and Children in Emergency Department and Urgent Care Settings. *Academic emergency medicine : official journal of the Society for Academic Emergency Medicine.* 2019. 26:719-731 |
| **Yang,​ Lianping,​Liu,​ Chaojie,​Wang,​ Lijun,​Yin,​ Xi,​Zhang,​ Xinping**. Public reporting improves antibiotic prescribing for upper respiratory tract infections in primary care: a matched-pair cluster-randomized trial in China. *Health research policy and systems.* 2014. 12:61 |
| **Yip,​ Winnie,​Powell-Jackson,​ Timothy,​Chen,​ Wen,​Hu,​ Min,​Fe,​ Eduardo,​Hu,​ Mu,​Jian,​ Weiyan,​Lu,​ Ming,​Han,​ Wei,​Hsiao,​ William C.**. Capitation combined with pay-for-performance improves antibiotic prescribing practices in rural China. *Health affairs (Project Hope).* 2014. 33:502-10 |
| **Zeiger,​ R. S.,​Schatz,​ M.,​Li,​ Q.,​Solari,​ P. G.,​Zazzali,​ J. L.,​Chen,​ W.**. Real-time asthma outreach reduces excessive short-acting β2-agonist use: a randomized study. *J Allergy Clin Immunol Pract.* 2014. 2:445-456,​ 456.e1-5 |
| **Zwar,​ N.,​Wolk,​ J.,​Gordon,​ J.,​Sanson-Fisher,​ R.,​Kehoe,​ L.**. Influencing antibiotic prescribing in general practice: a trial of prescriber feedback and management guidelines. *Family practice.* 1999. 16:495-500 |
